# Nanobodies against Cavin1 reveal structural flexibility and regulated interactions of its N-terminal coiled-coil domain

**DOI:** 10.1242/jcs.263756

**Published:** 2025-04-28

**Authors:** Ya Gao, Vikas A. Tillu, Yeping Wu, James Rae, Thomas E. Hall, Kai-En Chen, Saroja Weeratunga, Qian Guo, Emma Livingstone, Wai-Hong Tham, Robert G. Parton, Brett M. Collins

**Affiliations:** ^1^ Institute for Molecular Bioscience, The University of Queensland, St. Lucia, Queensland 4067, Australia; ^2^ Infectious Diseases and Immune Defence Division, The Walter and Eliza Hall Institute of Medical Research, Parkville, Victoria 3052, Australia; ^3^ Centre for Microscopy and Microanalysis, The University of Queensland, St. Lucia, Queensland 4067, Australia

**Keywords:** Caveolae, Caveolin, Cavin, PTRF, Nanobody

## Abstract

Caveolae are abundant plasma membrane structures that regulate signalling, membrane homeostasis and mechanoprotection. Their formation is driven by caveolins and cavins and their coordinated interactions with lipids. Here, we developed nanobodies against the trimeric HR1 coiled-coil domain of Cavin1. We identified specific nanobodies that do not perturb Cavin1 membrane binding and localise to caveolae when expressed in cells. The crystal structure of a nanobody–Cavin 1 HR1 complex reveals a symmetric 3:3 architecture as validated by mutagenesis. In this structure, the C-terminal half of the HR1 domain is disordered, suggesting that the nanobody stabilises an open conformation of Cavin1, which has previously been identified as important for membrane interactions. A phosphomimic mutation in a threonine–serine pair proximal to this region reveals selective regulation of Cavin2 and Cavin3 association. These studies provide new insights into cavin domains required for assembly of multiprotein caveolar assemblies and describe new nanobody tools for structural and functional studies of caveolae.

## Teaser

**Summary:** Structural and functional analyses of the binding between two nanobodies and the Cavin1 coiled-coil domain provide insights into cavin protein assemblies and caveola structures in cells.

## INTRODUCTION

The nanoscale (∼70 nm) cell-surface invaginations of the plasma membrane called caveolae (‘little caves’) are abundant in many vertebrate cells including endothelial cells, cardiac muscle fibers and adipocytes (Lamaze et al., 2017; Parton, 2018; Parton et al., 2020b). Caveolae possess a distinct protein and lipid composition consistent with their roles in physiological processes including endocytosis (Boucrot et al., 2011), regulation of intracellular signalling (Couet et al., 1997; Garcia-Cardena et al., 1997), lipid homeostasis (Harder and Simons, 1997; Kenworthy et al., 2023; Zhou et al., 2021) and mechanoprotection (Dewulf et al., 2019; Gervasio et al., 2011; Lo et al., 2015; Lolo et al., 2022; Lundmark et al., 2024; Sens and Turner, 2006; Sinha et al., 2011). Caveola dysfunction or loss has been implicated in a range of human diseases including cardiovascular arrhythmia (Campostrini et al., 2017; Fridolfsson and Patel, 2013; Jia and Sowers, 2015; Vatta et al., 2006), lipodystrophy and muscular dystrophy (Hayashi et al., 2009; Parton, 2018; Parton et al., 2020a; Rajab et al., 2010), highlighting their importance in animal physiology. Lipids and proteins act together to shape the plasma membrane-associated caveolae, which requires the assembly of two distinct protein families, caveolins and cavins, and their coordinated interactions with membrane lipids and cholesterol (Kenworthy et al., 2023; Ludwig et al., 2013; Lundmark et al., 2024; Parton et al., 2020b; Rothberg et al., 1992; Tillu et al., 2021; Zhou et al., 2021).

The integral membrane caveolins (CAV1, CAV2 and muscle-specific CAV3) are synthesised as 8S-CAV oligomers at the endoplasmic reticulum, which are then trafficked to the plasma membrane via the Golgi apparatus forming higher order oligomeric assemblies (Busija et al., 2017; Hayer et al., 2010; Morales-Paytuvi et al., 2023). CAV1 is sufficient to generate membrane invaginations in bacterial model systems that have the same size as typical caveolae in mammalian cells (Walser et al., 2012), and 11 CAV1 subunits are organised as a homo-oligomer in a spiralling flat disc that sits in the inner leaflet of the plasma membrane (Porta et al., 2022). However, in mammalian cells, CAV1 requires the presence of cavins to generate morphological caveolae (Hansen et al., 2009; Hayer et al., 2010; Hill et al., 2008; Liu and Pilch, 2008). The cavins are peripheral membrane proteins that form a multiprotein complex constitutively assembled in the cytosol and subsequently associated with 8S-CAV1 complexes at the plasma membrane (Bastiani et al., 2009; Hill et al., 2008). There are four different isoforms of cavin proteins in mammalian cells, Cavin1–Cavin4, which exhibit distinctive tissue-specific roles (Parton et al., 2020a). Cavin1 is ubiquitously expressed and is essential for stabilizing the caveolar domain and promoting the formation of the invaginated caveola structure (Hill et al., 2008). Cavin2 and Cavin3 compete with one another for interacting with Cavin1 to form higher-order hetero-oligomeric complexes that regulate the size and function of the caveola coat (Bastiani et al., 2009; Gambin et al., 2013; Ludwig et al., 2013; McMahon et al., 2019; Mohan et al., 2015). Cavin4 is a muscle-specific isoform.

All cavin proteins share a common primary structure possessing two highly conserved positively charged regions, helical regions (HRs) 1 and 2 (HR1 and HR2), separated by three poorly conserved disordered regions (DRs; DR1, DR2 and DR3), along their length (Burgener et al., 1990; Gustincich and Schneider, 1993; Hill et al., 2008; Kovtun et al., 2014; Tillu et al., 2018). The highly basic HR domains and uniformly acidic DR domains presumably produce a highly polarised cavin protein molecule (Bastiani et al., 2009). The molecular mechanism of caveola biogenesis is rapidly emerging from structural studies (Lundmark et al., 2024), including cryo-electron microscopy structures of CAV1 (Han et al., 2024 preprint; Porta et al., 2022) and crystal structures of Cavin1 and Cavin4 HR1 domains (Kovtun et al., 2014). The cavin HR1 domains form a highly extended trimeric coiled-coil assembly and provide a core structure thought to mediate intermolecular interactions of cavins promoting homo- and hetero-oligomerisation. A positively charged basic surface towards the C-terminus of the HR1 domain interacts preferentially with plasma membrane-enriched phosphatidylinositol 4,5-bisphosphate for lipid-dependent recruitment of cavins to caveolar membranes and membrane-remodelling activity (Hansen et al., 2009; Kovtun et al., 2014; Zhou et al., 2021). Notably, this region of the HR1 domain has also been found to undergo local unfolding during membrane interaction, leading to partial insertion of hydrophobic residues (Liu et al., 2022). The C-terminal HR2 domain of Cavin1 contains an 11-residue repeated amino acid sequence, termed the undecad repeat (UC1), that further promotes efficient binding with phosphatidylserine for membrane remodelling (Kovtun et al., 2014; Tillu et al., 2018). The HR2 domain is also functionally important for the self-assembly of cavins into larger oligomers and, together with HR1, associates with anionic lipid membranes (Kovtun et al., 2014). The three intrinsically unstructured DR domains of Cavin1 are strictly required for formation of caveola invagination and dynamic trafficking of caveolae (Tillu et al., 2021). Therefore, the assembly of caveolae has been proposed to depend on the low-affinity electrostatic interaction between CAV1 and Cavin1 as well as membrane lipid interactions (Kenworthy et al., 2023; Tillu et al., 2021).

With the aim of developing novel tools and reagents to study cavin structure and function, we turned to nanobodies. These single-domain antibodies derived from camelid species (e.g. alpacas and llamas) have allowed the development of new therapeutics and research tools for selective detection of proteins in their native environment, with advantages over antibodies or small molecules due to their high affinity, stability, improved solubility and yield (Jin et al., 2023). Using the mouse Cavin1 (mC1) HR1 complex, we immunised alpacas and subsequently isolated two high-affinity nanobodies, NbA12 and NbB7, that specifically target this Cavin1 coiled-coil domain. Crystal structures and mutagenesis define their binding epitopes within the N-terminal half of the HR1 domain. Interestingly, in the crystal structure of the NbB7–Cavin1 HR1 complex, the C-terminal region of the HR1 domain is disordered, suggesting that the nanobody is bound to a conformation that resembles the proposed semi-unfolded state of Cavin1 induced upon membrane binding. We further show that although NbA12 does not associate with caveolae in cells, NbB7 is robustly recruited to endogenous caveolae in a Cavin1-dependent manner. The unfolding of Cavin1 detected with NbB7 occurs at a central hydrogen-bonded pair of residues within the coiled-coil sequence. Mutagenesis of this region showed that it is critical for specificity between Cavin2 and Cavin3 recruitment in cells. These nanobodies thus appear to recognise epitopes that are differentially accessible *in vitro* and *in vivo*, and can be used as selective tools for structural analyses and cellular imaging of caveola assemblies.

## RESULTS

### Identification of highly potent nanobodies targeting Cavin1

**Fig. 1.**
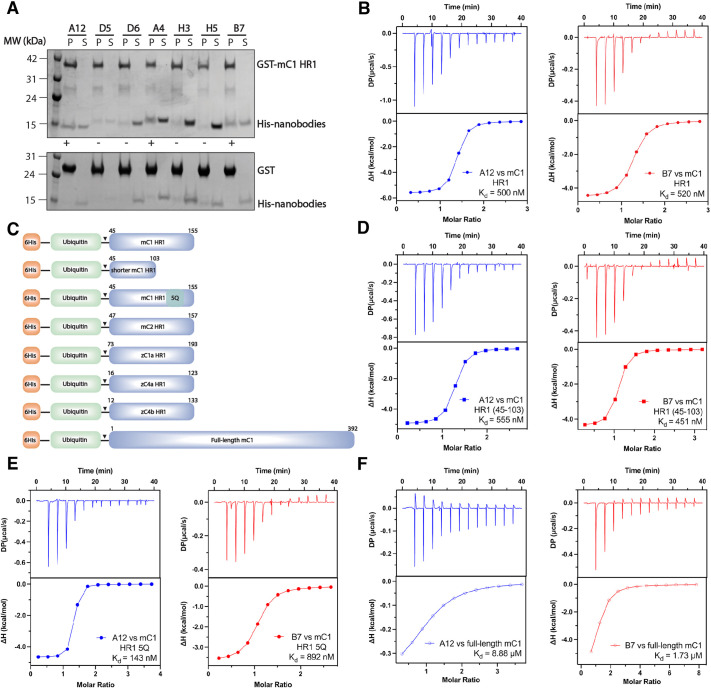
**Identification of Cavin1-binding nanobodies.** (A) Initial selections of nanobodies interacting with the mouse Cavin1 (mC1)-HR1 domain. GST–mC1-HR1 was used as a bait for His-tagged nanobodies. P, pellet; S, supernatant. (B) Isothermal calorimetry (ITC) thermogram for binding of nanobodies NbA12 and NbB7 to mC1-HR1. (C) Schematic representation of the HR1 domain and mutations in cavin family proteins tested for nanobody binding (shown to scale). (D) NbA12 and NbB7 showed identical binding affinity with an N-terminal fragment of mC1-HR1 (residues 45–103) as measured by ITC. (E) Similarly, both NbA12 and NbB7 interacted with the 5Q mutant of the mC1-HR1 domain as measured by ITC. (F) Binding of NbA12 and NbB7 with the full-length mC1 protein was still detected by ITC, although the affinity was reduced compared to that of the isolated HR1 domain. For all ITC graphs, the upper panel represents raw data, and the lower panel represents the normalised and integrated binding isotherms fitted with a 1:1 binding ratio. DP, differential power; ΔH, enthalpy of binding. The binding affinity (*K*_d_) is determined by calculating the mean of at least two independent experiments. Thermodynamic parameters for the binding analysis are provided in Table S1.

Nanobodies against the folded trimeric coiled-coil HR1 domain of mouse Cavin1 (mC1-HR1; residues 45–155) were produced by immunisation of an alpaca with the purified HR1 domain. Using a nanobody phage display library, we identified nanobodies that bound conformational epitopes of properly folded HR1 domain. From 43 positive phage supernatants that recognised the mC1-HR1 domain by preliminary enzyme-linked immunosorbent assay (ELISA) screening, we identified seven distinct clonal groups based on unique CDR3 sequences, and expressed and purified one representative nanobody from each clonal group for further characterisation. Of these nanobodies, NbA12 and NbB7 were found to show the strongest interaction with the mC1-HR1 domain by GST pulldown assay, with NbA4 showing a potential weaker interaction (Fig. 1A). These were then validated by isothermal titration calorimetry (ITC), and NbA12 and NbB7 were both found to bind purified mC1-HR1 with nanomolar affinities (Fig. 1B; Table S1). In contrast, NbA4 did not show any substantial binding affinity by ITC (Fig. S1A). The sequences of NbA12 and NbB7 are provided in Fig. S1B.

To map the binding regions of the nanobodies, we tested a series of mC1-HR1 truncation and mutant constructs (Fig. 1C). The N-terminal fragment mC1-HR1(45–103) retains the stable trimeric coiled-coil conformation when expressed and purified from *Escherichia coli* (Kovtun et al., 2014) and displayed an almost identical binding affinity for both NbA12 and NbB7 nanobodies (Fig. 1D) as the full HR1 domain (Fig. 1B,D). This indicates that C-terminal region of the HR1 domain is not required for binding with these nanobodies. Previously, substitutions of several key charged arginine and lysine residues (Lys115, Arg117, Lys118, Lys124 and Arg127) with glutamine residues located in a basic cluster at the C-terminus of the Cavin1 HR1 domain were made (5Q mutant), which disrupt its association with negatively charged phospholipid membranes but still maintain proper helical structure (Kovtun et al., 2014). Consistent with binding of the N-terminal region of the HR1 domain, we found that the binding affinities of the 5Q mutant for the nanobodies NbA12 and NbB7 were not substantially different to those of the wild-type HR1 domain (Fig. 1E).

Next, we tested binding of NbA12 to mC1-HR1 in the presence of a molar excess of NbB7 and found that binding was no longer detectable (Fig. S1C). This suggests that the nanobodies most likely share an overlapping epitope on the HR1 coiled-coil structure. Lastly, we tested these two nanobodies against full-length MBP-tagged mC1. Notably, although both nanobodies still showed substantial interactions, they were found to have reduced binding affinities compared to those with the isolated HR1 domain alone (Fig. 1F; Table S1). The full-length mC1 protein is relatively difficult to purify compared to the HR1 domain alone, but we previously showed it can also form larger molecular mass oligomers of the core Cavin1 trimer (Kovtun et al., 2014; Tillu et al., 2021). We speculate that potential intramolecular and/or intermolecular interactions of the HR1 domain with other regions of the Cavin1 protein might be partially occluding the nanobody-binding site.

Given the high affinity of both nanobodies for the mC1-HR1 domain immunogen, we then sought to test the interaction of nanobodies with the HR1 domain in other cavin proteins to examine its binding specificity. NbA12 and NbB7 both showed binding with the mouse Cavin2 HR1 (mC2-HR1) domain but with much lower affinity (*K*_d_) in the range of 3.4–7.7 μM (Fig. S1D). Next, we also tested the binding of the nanobodies with cavin homologues from zebrafish to see if they showed cross-species reactivity. A similar micromolar binding affinity result was detected where we observed binding between both nanobodies and the Cavin1a HR1 domain from zebrafish (zC1a-HR1) (Fig. S1E). Zebrafish Cavin4a HR1 (zC4a-HR1) has been confirmed to form a similar trimeric coiled-coil structure by X-ray crystallography (Kovtun et al., 2014). In contrast to the nanobody NbB7, which showed a weak binding in the micromolar range, the nanobody NbA12 was unable to bind to zC4a-HR1 (Fig. S1F). For zebrafish Cavin4b, these two nanobodies showed no interaction (Fig. S1G). In summary, these two nanobodies are relatively specific for mouse Cavin1, but possess some weak cross reactivity with mouse Cavin2 and with cavin homologues in other species.

### Cavin1 retains lipid binding and remodelling activity in the presence of nanobodies

**Fig. 2.**
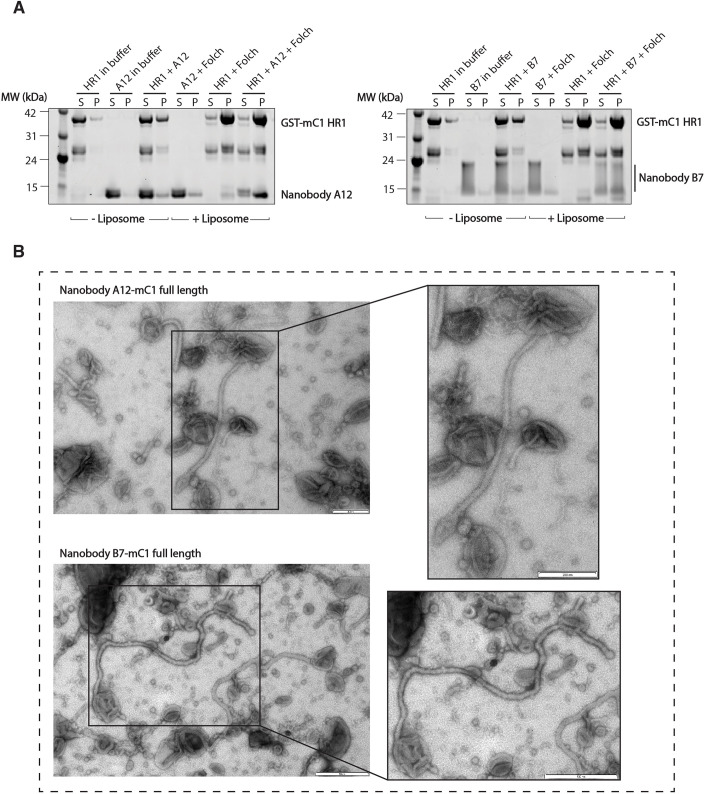
**Nanobodies do not affect the *in vitro* intrinsic membrane-remodelling properties of Cavin1.** (A) Liposome pelleting assay of the mouse Cavin1 (mC1)-HR1 domain in the presence of nanobodies. Multilamellar vesicles were generated from Folch I lipids. In the presence or absence of NbA12 or NbB7, the mC1-HR1 domain was incubated with or without liposomes and centrifuged. S, unbound supernatant; P, bound pellet. Images represent two independent experiments. (B) Negatively stained electron microscopy images of nanobody–Cavin1 complexes incubated with Folch I liposomes. His-MBP full-length mC1 was used to test liposome tubulation in the presence of either NbA12 or NbB7. Images represent two independent experiments. Scale bars: 200 nm (top, NbA12–mC1); 500 nm (bottom, NbB7–mC1).

The Cavin1 HR1 domain was previously shown to interact with negatively charged phospholipid membranes, promoting recruitment of cavins to caveolar membranes and membrane-remodelling activity *in vitro* (Kovtun et al., 2014). We first tested whether the nanobodies affected membrane binding by the HR1 domain using artificial liposomes in pelleting assays. For this experiment, we used liposomes composed of bovine brain-derived Folch fraction, which is highly enriched in negatively charged phospholipids. As shown previously (Kovtun et al., 2014), using GST-tagged mC1-HR1, we confirmed a high-affinity association with Folch liposomes (Fig. 2A). This interaction was not affected by either NbA12 or NbB7, which were both co-pelleted with the GST-tagged mC1-HR1. Full-length Cavin1 was also shown to have an ability to remodel and tubulate artificial liposomes *in vitro* (Kovtun et al., 2014). As shown in Fig. 2B, the nanobodies did not perturb the ability of full-length recombinant mC1 to remodel and tubulate artificial liposomes *in vitro*. Altogether, these results indicate that the binding of the nanobodies does not inhibit the key membrane-interacting activities of Cavin1.

### NbB7 labels Cavin1-coated caveolae in cells

**Fig. 3.**
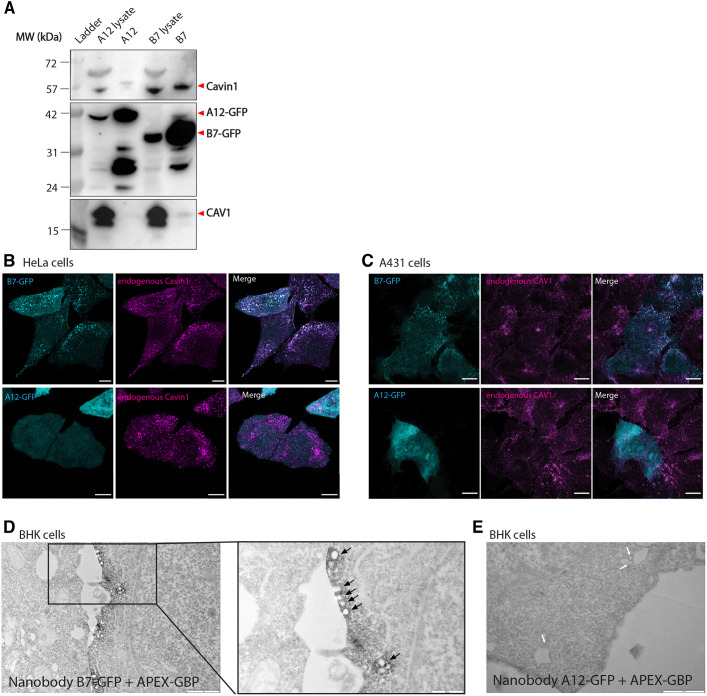
**NbB7 can be used as a molecular probe for Cavin1 in cells.** (A) Co-immunoprecipitation of GFP-tagged nanobodies with Cavin1 and CAV1. HeLa cells expressing NbA12–GFP or NbB7–GFP were incubated with a GFP-binding nanobody coupled to NHS-activated Sepharose resin. Cavin1 and CAV1 were detected by western blotting. Blots represent two independent experiments. (B) Confocal microscopy of HeLa cells transfected with NbB7–GFP (top) and NbA12–GFP (bottom) nanobodies. Fixed cells were also stained for Cavin1. Scale bars: 10 μm. (C) Confocal microscopy of A431 cells transfected with NbB7–GFP (top) and NbA12–GFP (bottom) nanobodies. Fixed cells were also stained for CAV1. Scale bars: 10 μm. Fluorescence images represent two independent experiments. (D) The NbB7–GFP nanobody was detected at the cell plasma membrane by electron microscopy of BHK cells co-transfected with constructs expressing APEX–GBP and NbB7–GFP. Black arrows represent invaginated caveolae labelled with APEX–GBP. Scale bars: 1 μm (left); 500 nm (right expanded view). (E) The NbA12–GFP nanobody showed cytosolic labelling by electron microscopy of BHK cells co-transfected with constructs expressing APEX–GBP and NbA12–GFP. White arrows indicate caveolae without APEX–GBP labelling. Scale bar: 1 μm. Electron micrographs represent a single experiment.

As both nanobodies NbA12 and NbB7 can bind Cavin1 *in vitro* and do not perturb its ability to associate with membranes, we next tested their capacity to label Cavin1 in several different cell lines, including human HeLa and A431 cells and baby hamster kidney (BHK) cells. Their interaction with the two structural proteins of caveolae, Cavin1 and CAV1, were first validated by immunoprecipitation in transiently transfected HeLa cells. Lysates from cells expressing C-terminal GFP-tagged NbA12 (NbA12–GFP) and NbB7 (NbB7–GFP) were incubated with a GFP-binding nanobody (Kubala et al., 2010) covalently coupled to NHS-activated Sepharose resin. Under these co-immunoprecipitation conditions with mild detergent (0.5% Triton X-100), only NbB7–GFP could co-precipitate endogenous Cavin1 and, to a lesser extent, CAV1 (Fig. 3A). One important note is that, for unknown reasons, GFP-tagged NbB7 and NbA12 nanobodies migrated at different molecular masses by SDS-PAGE. This experiment was repeated several times with similar results, and plasmids were re-sequenced to confirm that there were no differences at the nucleotide levels for these constructs. In A431 cells, which had lower expression levels of the nanobodies, we could detect NbB7–GFP at the correct molecular mass with an additional band at a smaller size, suggesting that some truncation or nicking of the protein might be occurring (Fig. S1H).

We next investigated the co-localisation of nanobodies with Cavin1 and CAV1 using an orthogonal set of cells by fluorescence microscopy. HeLa and A431 cells express both endogenous Cavin1 and CAV1 and form typical caveola puncta at the cell plasma membrane. Consistent with the co-immunoprecipitation results, we observed a high degree of co-localisation between NbB7–GFP and endogenous Cavin1 at the plasma membrane of HeLa cells, whereas in contrast, NbA12–GFP was diffusely expressed in the cytoplasm (Fig. 3B). The NbB7–GFP nanobody also showed a high degree of co-localisation with endogenous CAV1 in A431 cells, with a characteristic punctate caveola distribution, whereas the NbA12–GFP nanobody again showed no co-localisation with CAV1 (Fig. 3C). The lack of Cavin1 or caveola association of NbA12 is consistent with its poor binding in immunoprecipitation experiments and suggests that, although NbA12 can bind Cavin1 *in vitro*, its required binding epitope may not be accessible in the membrane-assembled caveola complex.

As NbB7–GFP showed clear association with caveolae by fluorescence imaging, we next examined its localisation by electron microscopy. The APEX tag, derived from soybean ascorbate peroxidase, has been developed as a fast, sensitive and reliable labelling system by fusing it to a GFP-binding peptide (GBP) that allows direct imaging of target proteins fused to a GFP tag (Ariotti et al., 2015). Here, we co-transfected BHK cells with NbB7–GFP and APEX–GBP in cells. NbB7–GFP labelling with APEX–GBP revealed an electron-dense reaction product strictly associated with caveolae at the cell plasma membrane (Fig. 3D). The co-expression of NbA12–GFP and APEX–GBP resulted in a cytosolic labelling with no visible caveolae labelled (Fig. 3E). This high-resolution protein localisation analysis indicates that the nanobody NbB7 is highly suitable for use as a bioactive tool to explore cavins and caveolae in cells.

### NbB7 binding promotes partial unfolding of the HR1 coiled-coil structure

**Fig. 4.**
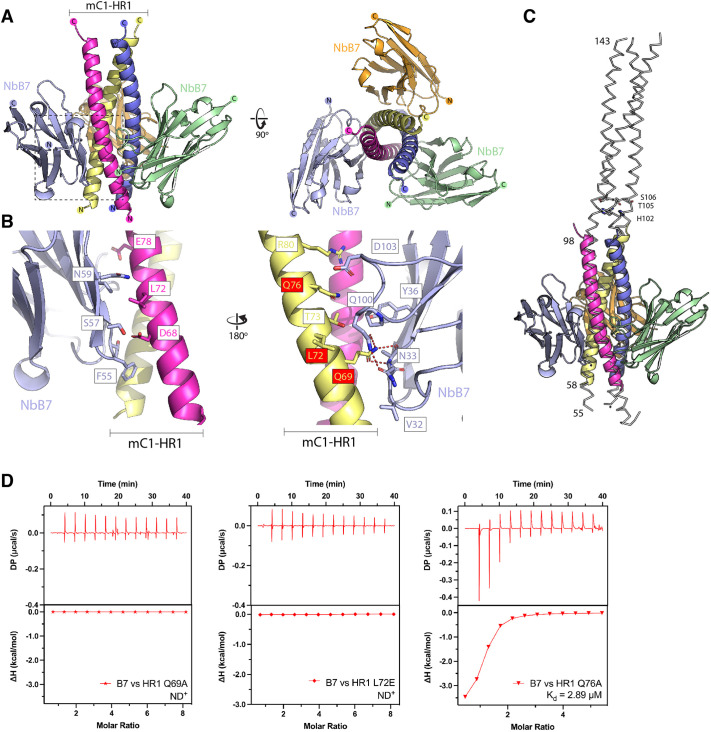
**Molecular basis of nanobody NbB7 interaction with the mouse Cavin1 HR1 domain.** (A) The crystal structure of trimeric mouse Cavin1 (mC1)-HR1 in complex with NbB7. The asymmetric unit contains one copy of the mC1-HR1 protein and one copy of NbB7, with the trimeric structure inferred from the crystallographic symmetry. (B) Close-up of the boxed region in A highlighting key residues mediating the interaction between NbB7 (violet) and two adjacent helices of the mC1-HR1 coiled coil (magenta and yellow). Residues in mC1-HR1 tested by mutagenesis are boxed in red. (C) Comparison of the mC1-HR1–NbB7 complex (coloured cartoon) with the previous crystal structure of the mC1-HR1 domain (PDB ID: 4QKV) (Kovtun et al., 2014). Numbers on the left indicate N- and C-terminal residues of mC1-HR1 visible in the electron density for the NbB7–mC1-HR1 complex (residues 55–98) and for mC1-HR1 alone (residues 55–143). Residues His102, Thr105 and Ser106 mutated in later experiments are indicated on the right. (D) ITC binding experiments of NbB7 and selected mutations in mC1-HR1. ND^+^, not determined.

We next aimed to characterise the molecular basis of nanobody binding to Cavin1. We co-crystallised the mC1-HR1 trimeric coiled-coil domain with NbA12 and NbB7. Attempts to obtain diffraction-quality crystals with NbA12 were unsuccessful; however, a stable stoichiometric complex of the nanobody NbB7 and mC1-HR1 purified by size-exclusion chromatography was crystallised by sparse-matrix screening and optimisation. The structure of the NbB7–mC1-HR1 complex was solved by X-ray crystallography at 1.5 Å resolution. This revealed a symmetric complex with three copies of NbB7 bound to the mC1-HR1 trimer, with trimeric symmetry along the crystallographic 3-fold axis. The structure showed clear electron density for NbB7 interacting with the N-terminal region of the trimeric coiled-coil HR1 residues 56–98 (or residues 45–155 in the crystallised construct) (Fig. 4A–C; Table S2). One notable feature of the structure, however, was that when the Cavin1 HR1 protein formed a complex with NbB7, the C-terminal half of the HR1 coiled-coil appeared to have unravelled, with no visible electron density for HR1 residues 99–155. It is clear that if this region formed an extended coiled coil as previously observed for apo-mC1 and zC4 HR1 domain structures (Kovtun et al., 2014), it would not be compatible with the packing of the crystal lattice. Unfolding of the C-terminus of the HR1 domain was recently proposed to be important for membrane interaction (Liu et al., 2022) and is discussed further below.

Each copy of NbB7 interacted with two adjacent protomers of the mC1-HR1 coiled-coil trimer (Fig. 4B). The interaction was, as expected, predominantly mediated by the three CDR loops of the nanobody, but also involved contacts with residues along the faces of the β3, β4 and β5 strands of the nanobody structure. Several hydrophobic contacts helped to stabilise the interaction, including Leu72 of mC1-HR1 and Phe55 in NbB7 (within the CDR2 loop). One key contact involved Gln67 of mC1-HR1, which formed a network of hydrogen bonds with the nanobody, including with Asn33 in the CDR1 loop and Gln100 in the CDR3 loop. To validate the binding mechanism, we generated several single-site mutations in mC1, including Q69A, L72E and Q76A, and tested their impact on binding by ITC (Fig. 4D; Table S3). Whereas the Q76A mutation in HR1 substantially reduced the binding affinity for the nanobody NbB7 to a micromolar level, both Q69A and L72E mutations reduced the binding to undetectable levels.

**Fig. 5.**
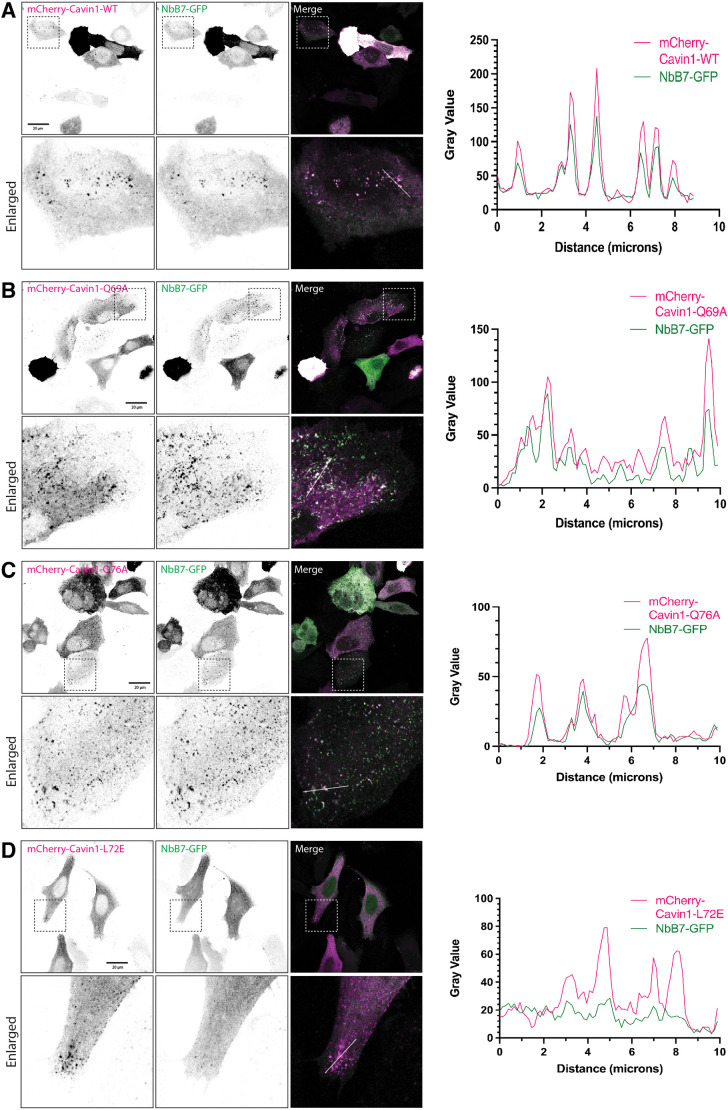
**Mutational analysis of the NbB7–Cavin1 interaction in cells.** (A–D) Confocal images showing the localisation of transiently co-expressed NbB7–GFP nanobody with wild type (WT) or mutated mCherry–Cavin1 in HeLa Cavin1 knockout cells. Images for each channel were inverted into black and white. The bottom panels display enlarged views of the selected regions indicated by dashed boxes in the top panels. Scale bars: 20 μm. Co-localisation between NbB7–GFP and mCherry–Cavin1 was examined by line profile analysis. Scanned lines are indicated in white in the merged images. Images represent two independent experiments.

To confirm that the structurally mapped sites are responsible for the co-localisation with Cavin1 in cells, re-expression of mCherry–Cavin1 in the CRISPR-generated Cavin1 knockout HeLa cells rescued the co-localisation of nanobody NbB7 to the plasma membrane (Fig. 5A). As seen in Cavin1 knockout HeLa cells, the Cavin1 Q69A and Q76A mutants still co-localised with NbB7–GFP at the plasma membrane, forming the typical puncta (Fig. 5B,C). In contrast, the Cavin1 L72E mutant, which did not bind the nanobody *in vitro*, did not recruit NbB7–GFP for co-localisation (Fig. 5D). These findings further confirm that the leucine residue Leu72 of Cavin1 is crucial for the strong binding with the nanobody NbB7.

### Structural assessment of the role of a conserved hydrogen-bonded structure in Cavin1 HR1

**Fig. 6.**
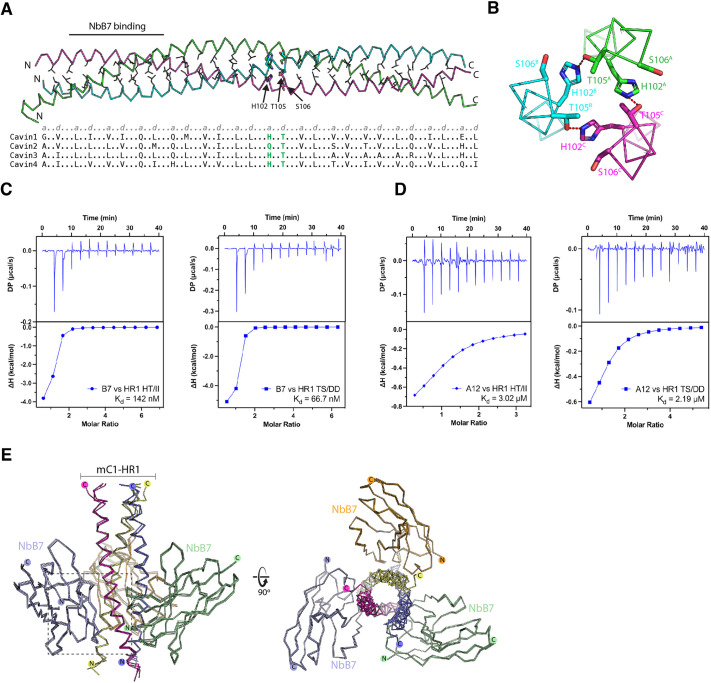
**Altered dynamics in binding affinity between nanobodies and hydrogen-bonded pair mutants in Cavin1.** (A) Heptad repeat sequence alignments of cavin proteins. The top panel represents the mouse Cavin1 (mC1) HR1 domain structure and the central residues are shown with sidechains. The bottom panel shows the sequence alignment of heptad repeats in human Cavin1–Cavin4 with the corresponding residues at positions *a* and *d*. The histidine–threonine (His102–Thr105 in mC1) pair is highlighted in green. (B) The hydrogen-bonded pair forms between the central His102–Thr105 sidechains in mC1. (C,D) The interactions of the nanobodies NbB7 (C) and NbA12 (D) with the mC1-HR1 HT/II and TS/DD mutants were validated by ITC. Upper panels represent raw data and lower panels represent the binding isotherms. The binding affinity (*K*_d_) was determined by calculating the mean of at least two independent experiments. (E) Structural alignment of the complexes formed by the mC1-HR1 wild-type protein or HT/II and TS/DD mutants with the nanobody NbB7.

The observation that the C-terminal half of the Cavin1 HR1 domain is apparently unfolded in the NbB7 co-crystal structure prompted us to investigate this further. There are two previous findings directly relevant to this: first, the HR1 domain can be divided into two halves based on a central hydrogen-bonded structure of the coiled coil (Kovtun et al., 2014) and, second, there is evidence for unravelling of the C-terminal portion of the HR1 domain upon membrane interaction (Liu et al., 2022). The HR1 domain forms a trimeric coiled coil that is ∼100 residues in length and is composed of typical heptad repeats, denoted *abcdefg*, where the *a* and *d* positions are generally hydrophobic amino acids, such as leucine, isoleucine and valine, required for packing of adjacent helices (Kovtun et al., 2015, 2014; Tillu et al., 2018) (Fig. 6A). However, at the centre of the HR1 domain in Cavin1 are a conserved pair of histidine and threonine residues (His102 and Thr105 in mC1; Fig. 6B) that form a buried hydrogen-bond interaction. We speculated that these could be analogous to the central arginine–glutamine pairing in the coiled-coil structures of the soluble SNARE complex (Scales et al., 2001; Sutton et al., 1998). That is, the histidine–threonine pair in Cavin1 helps to define the specificity of the homomeric and heteromeric protein–protein interactions with itself and Cavin2 and Cavin3 because it prevents binding of other heptad repeat-containing coiled-coil proteins, and might provide a point of instability that allows for regulated assembly and disassembly of the coiled coil (Kovtun et al., 2015). In addition, Thr104, Thr105 and Ser106 in mouse and human Cavin1 are reported phosphorylation sites in Phosphosite (Hornbeck et al., 2015), and insulin-stimulated phosphorylation of Thr104 and Ser106 has been reported (Humphrey et al., 2013). The Thr105–Ser106 pair was previously hypothesised to be able to disrupt the hydrogen-bonded pair buried within the coiled coil (Kovtun et al., 2015).

To investigate the importance of the His102–Thr105 and Thr105–Ser106 pairs, we designed two sets of mutations. We hypothesised that altering the His102–Thr105 pair in the mC1-HR1 domain to isoleucine sidechains (HT/II mutant) might stabilise this region of the coiled coil by converting these to typical hydrophobic sidechains of a coiled-coil heptad repeat. The second mutation was to make putative phosphomimetic alterations in the Thr105–Ser106 pair (TS/DD mutant) that we predicted could disrupt coiled-coil formation. Surprisingly, both mutant proteins could be purified similarly to the wild-type mC1-HR1 trimer and they did not display dramatic differences in stability when we examined their melting temperatures by differential scanning fluorimetry (Fig. S2). When we tested their interactions with NbB7, we found only small changes in their binding affinity and specificity (Fig. 6C,D; Table S4). The binding affinity (*K*_d_) of NbB7 for the mC1-HR1(HT/II) mutant was moderately enhanced from 520 to 142 nM, whereas its interaction with the mC1-HR1(TS/DD) mutant was improved by ∼8-fold with a *K*_d_ of 66.7 nM. In contrast to NbB7, although NbA12 could still bind the mC1-HR1(HT/II) and mC1-HR1(TS/DD) mutants, the affinities were significantly lower at 3 and 2 µM, respectively. This suggests that although NbA12 binds an epitope that overlaps with that bound by NbB7 (∼residues 65–85) (Fig. 1F), its binding extends further towards the C-terminus and encompasses the central His102–Thr105 region.

We determined the crystal structures of both the mC1-HR1(HT/II) and mC1-HR1(TS/DD) mutants in complex with NbB7, hoping to observe changes in the folding of the C-terminal region of the HR1 domain (Fig. 6E; Table S2). Interestingly, however, although both structures were obtained in different space groups to the wild-type protein, they still showed a lack of density for the C-terminal region of the mC1-HR1 domain beyond Lys97. In both cases, the C-terminal region including the mutation sites was, therefore, still disordered. This also suggests neither the HT/II nor TS/DD mutations prevented uncoiling of the C-terminal region when bound to NbB7 at the N-terminal region.

### Investigating the impact of polar sidechains on cavin–cavin interactions in cells

**Fig. 7.**
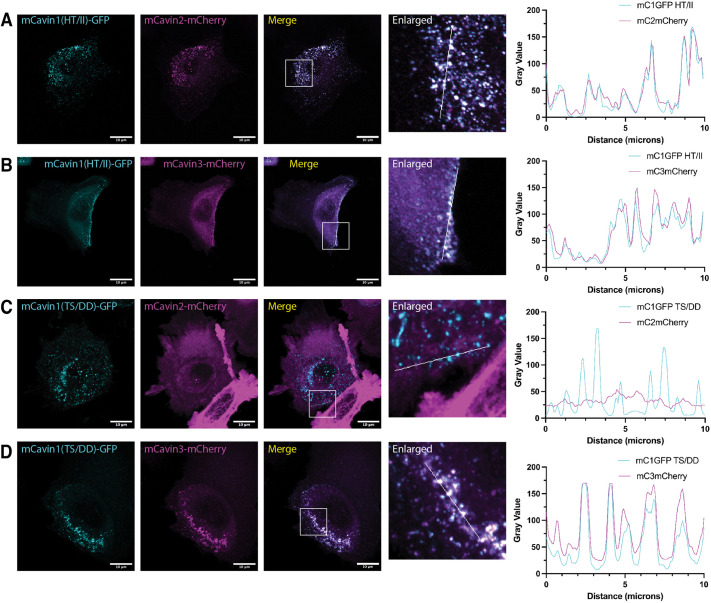
**Mutations in the Cavin1 HR1 domain inhibit recruitment of Cavin3 to caveolae.** (A–D) Confocal images showing the localisation of transiently co-expressed mouse Cavin1–GFP mutants with mouse Cavin2–mCherry or mouse Cavin3–mCherry in PC3 cells. These cells lack endogenous cavins and only form caveolae when Cavin1 is exogenously expressed. Scale bars: 10 μm. Co-localisation between Cavin1–GFP and mCherry-tagged proteins was examined by line profile analysis. Scanned lines are indicated in white in the enlarged images. Images represent two independent experiments.

Although the HT/II and TS/DD mutations had only limited impacts on mC1-HR1 homotrimer stability and folding *in vitro*, we discovered a striking phenotype when these mutations were introduced into full-length Cavin1 in cells. To assess whether the mutations impacted the ability of Cavin1 to assemble with caveolae or interact with other cavin family members, we co-transfected constructs expressing mC1–GFP mutants with either Cavin2–mCherry or Cavin3–mCherry in PC3 cells (Fig. 7). PC3 cells lack expression of any cavin family proteins and do not show morphological caveolae. Caveola formation can be rescued, however, by exogenous expression of Cavin1, which can recruit both Cavin2 and Cavin3 if they are also co-expressed (Bastiani et al., 2009; Hill et al., 2008). Similar to the wild-type Cavin1 protein (Bastiani et al., 2009; Hill et al., 2008), confocal microscopy showed that Cavin1(HT/II)–GFP and Cavin1(TS/DD)–GFP both localised to typical punctate caveola structures at the plasma membrane, indicating that the mutations do not prevent the ability of Cavin1 to form caveolae (with membrane-embedded CAV1). Intriguingly, however, although Cavin1(HT/II)–GFP could stably recruit both Cavin2–mCherry and Cavin3–mCherry to caveola puncta when co-expressed (Fig. 7A,B), the phosphomimic TS/DD mutant of Cavin1 recruited Cavin3–mCherry (Fig. 7D) but not Cavin2-mCherry, which remained dispersed in the cytosol (Fig. 7C). This striking observation indicates that the putative phosphorylation site in Cavin1 (Humphrey et al., 2013) can play a role in determining specific caveola recruitment of the Cavin2 and Cavin3 homologues.

## DISCUSSION

Cavin1 is an essential component of the caveola coat, interacting with the membrane-embedded CAV1 protein and required for the recruitment of Cavin2 and Cavin3 homologues to the site of caveola assembly (Bastiani et al., 2009; Gambin et al., 2013; Ludwig et al., 2013; McMahon et al., 2019; Mohan et al., 2015). Its sequence and structure is highly distinctive, with only the HR1 domain known to form an ordered structure in the shape of a membrane-binding trimeric coiled coil, whereas the rest of its sequence is either intrinsically disordered or only transiently folded when forming larger complexes (Kovtun et al., 2015, 2014; Liu et al., 2022; Tillu et al., 2018, 2021). Furthermore, molecular dynamics simulations have suggested that the HR1 domain itself also possesses structural plasticity, where its C-terminus can partially unfold to allow for hydrophobic residues to insert into the lipid bilayer (Liu et al., 2022; Lundmark et al., 2024). Although a detailed understanding of the organisation of the core Cavin1 and CAV1 proteins in the caveola coat remains elusive, the structures of the Cavin1 HR1 domain (Kovtun et al., 2014) and of homo-oligomeric CAV1 discs (Han et al., 2020) and studies of their interactions point towards a hierarchy of multivalent interactions between proteins and the lipid membrane driving caveola formation (Han et al., 2024 preprint; Kenworthy et al., 2023; Kovtun et al., 2015; Liu et al., 2022; Lundmark et al., 2024; Mohan et al., 2015; Tillu et al., 2018, 2021).

To develop new tools for functional analysis and structural studies of Cavin1, we characterised two nanobodies that bind specifically to the Cavin1 HR1 coiled coil. Nanobodies and their derivatives are rapidly emerging as powerful research tools for structural determination and cellular engineering in biological research, and as potential biologic therapeutics (Muyldermans, 2021; Uchański et al., 2020). The two nanobodies NbA12 and NbB7 each bound with similar affinities to the trimeric HR1 domain, with overlapping but not identical epitopes within the N-terminal half of the extended coiled-coil structure. They were relatively specific and bound to Cavin1 from zebrafish, although they showed some mild cross-reactivity with Cavin2 and Cavin4. In the presence of both nanobodies *in vitro*, Cavin1 was found to retain its inherent anionic membrane interaction and membrane remodelling ability. In the future, cryo-electron tomography of the membrane tubules generated by the Cavin1–nanobody complexes might reveal the precise mode of binding of the membrane by the Cavin1 protein and whether the presence of the nanobodies affects the conformation of Cavin1 upon membrane recognition.

The crystal structure of NbB7 in complex with mC1-HR1 revealed the precise mechanism of its interaction and allowed us to confirm the binding site via structure-based mutagenesis. However, a remarkable feature of this crystal structure was that the C-terminal portion of the coiled coil of HR1 (residues 98–155) was unexpectedly absent in the electron density compared to mC1-HR1 on its own (Kovtun et al., 2014). Based on considerations of the symmetry of the crystal lattice and the fact that structures of two other mC1-HR1 mutants in complex with NbB7 showed a similar lack of electron density, we are confident that this indicates that the C-terminal residues have become disordered due to unravelling of the coiled coil in this region. What does this mean? We believe that this points to an inherent instability in the HR1 coiled coil beyond the central hydrogen-bonded histidine and threonine residues, and that either upon binding of NbB7 at the N-terminus or during crystallisation of the complex, the C-terminal region can transition into an unfolded state or shifts its equilibrium between folding and unfolding. Although this remains to be categorically proven, the observation provides support for the previous modelling that indicates that the Cavin1 HR1 domain can partially unfold at its C-terminus when binding to a lipid membrane (Liu et al., 2022).

The discovery that the C-terminal half of the Cavin1 HR1 domain can adopt an unfolded state, made possible using NbB7, prompted us to examine the site of ‘uncoiling’ more closely. This region corresponds to the centre of the coiled coil defined by the hydrogen-bonded residues His102 and Thr105 (Liu et al., 2022), and a site of putative phosphorylation including Thr105 and Ser106, which is upregulated in insulin-stimulated adipocytes (Humphrey et al., 2013). We hypothesised that mutations at these sites act to either stabilise the HR1 coiled coil (substituting the buried hydrogen-bond pair with hydrophobic sidechains) or perturb its assembly (adding negative charges to the threonine and serine sidechains). These mutations did not dramatically impact the coiled-coil stability *in vitro* nor the ability of the full-length Cavin1 protein to promote caveola formation in cells. However, without affecting Cavin3, the phosphomimetic mutations in Cavin1 led to a complete inability to recruit the Cavin2 homologue to caveolae. Previous studies have shown that Cavin1 forms distinct complexes with either Cavin2 or Cavin3 (Bastiani et al., 2009; Gambin et al., 2013; Ludwig et al., 2013; McMahon et al., 2019; Mohan et al., 2015) and that Cavin3 can be specifically released from caveolae upon cellular stress (McMahon et al., 2021). Our results show that sequences within the central region of the HR1 domain can define specificity for Cavin3 over Cavin2 and, potentially, that post-translational modification of Cavin1 at this site is important for regulating these specific interactions in the cell. This raises the fascinating possibility that the cavin complement, and presumably function, of caveolae can be dynamically modulated by post-translational modifications *in vivo* in response to specific physiological signals.

This work provides the first report of structurally characterised nanobody probes that can be used as molecular tools for studying caveola-associated proteins. These nanobodies have provided novel insights into the folding and stability of the Cavin1 HR1 domain, and NbB7 can be used for the imaging of Cavin1 and caveolae in cells by fluorescence and electron microscopy and for imaging Cavin1 *in situ* and *in vivo.* For example, in other work, we recently applied NbB7 to quantify changes in caveola numbers under different lipid peroxidation conditions (Wu et al., 2024 preprint). Future applications could include use as affinity reagents for proteomics analyses of endogenous Cavin1, altering Cavin1 activity via overexpression or targeting of modifying enzymes, use as fiducial markers in cryo-electron tomography studies of Cavin1-coated membranes and vesicles, studying the dynamics of caveolae by high-resolution caveola tracking in cells, or for correlative light and electron microscopy imaging of caveolae to answer many long-standing mysteries about their organisation.

## MATERIALS AND METHODS

Details of key resources, including bacterial strains, chemicals and software, are provided in Table S6.

### Molecular cloning and plasmids

All plasmids are listed in Table S5. For bacterial expression of cavin proteins and their helical domains, DNA encoding N-terminal 6× histidine–ubiquitin (His–Ub)-tagged mouse Cavin1 (mC1)-HR1, shorter fragment of mC1-HR1 (residues 45–103), mC1 HR1 5Q mutant, mouse Cavin2 (mC2)-HR1, zebrafish Cavin1a (zC1a)-HR1, zebrafish Cavin4a (zC4a)-HR1, zebrafish Cavin4b (zC4b)-HR1, and single-site mutants of mC1-HR1 (Q69A, L72E and Q76A) were all cloned into the pHUE vector (NovaPro, V010154) at the SacII restriction enzyme site using overlap extension-polymerase chain reaction (OE-PCR) (Catanzariti et al., 2004). DNA encoding mC1 full-length was cloned into the 2K-T vector (Addgene plasmid 37183) containing a His-MBP tag at the SspI restriction enzyme site using OE-PCR. Constructs encoding the N-terminal GST-tagged mC1-HR1 and H102I/T105I (HT/II) and T105D/S106D (TS/DD) mutants were cloned into the pGEX-4T2 vector (Cytiva) with a site-specific thrombin cleavage sequence using OE-PCR. For mammalian cell expression, constructs encoding the nanobodies NbA12 and NbB7 and mC1 full-length point mutants Q69A, L72E, Q76A, HT/II and TS/DD were cloned into the pEGFP-N1 vector (NovaPro, V012021) with a C-terminal GFP tag at the BamHI restriction enzyme site. Cavin2–mCherry and Cavin3–mCherry constructs were reported previously (Gambin et al., 2013). Site-directed mutagenesis was applied to all mutant constructs with custom-designed primers. The GFP-binding nanobody was expressed and purified as described previously (Kubala et al., 2010).

### Recombinant protein expression and purification of cavin proteins

N-terminal His-Ub-tagged mC1-HR1, the shorter fragment of mC1-HR1 (residues 45–103), the mC1-HR1 5Q mutant, mC2-HR1, single-site mutants of mC1-HR1 (Q69A, L72E and Q76A) and His-MBP-tagged full-length mC1 were transformed into *E. coli* BL21(DE3)pLysS competent cells by heat shock treatment at 42°C. The codon-optimised zC1a-HR1, zC4a-HR1 and zC4b-HR1 constructs and N-terminal GST-tagged mC1-HR1 and mC1-HR1 HT/II and TS/DD mutants were transformed into *E. coli* BL21 CodonPlus (DE3) cells. Cells were propagated in fresh LB broth at 37°C at 200 rpm until the optical density at 600 nm reached 0.8–1.0, and cells were then induced with 0.5 mM isopropyl β-D-1-thiogalactopyranoside (IPTG) at 18°C for 16 h. Cells were harvested and lysed using a continuous flow cell disruptor (Constant Systems Limited, UK) at 170–220 kPa in the GF500 buffer containing 20 mM HEPES at pH 7.4 and 500 mM NaCl with 50 μg/ml benzamidine hydrochloride, 10 μg/ml deoxyribonuclease I (DNase I), 0.5% w/v Triton X-100 and 5 mM imidazole (for His-Ub-tagged cavins only), followed by high-speed centrifugation at 38,000 ***g*** for 30 min. All recombinant proteins with a His-Ub tag and His-MBP full-length mC1 were purified by TALON metal affinity resin (Clontech, Scientifix #635503) and washed with GF500 containing 5 mM imidazole to remove contaminants (used for ITC experiments). Protein samples were then eluted with GF500 containing 300 mM imidazole. GST-tagged mC1-HR1 and the HT/II and TS/DD mutants were purified by glutathione Sepharose affinity resin (GE healthcare), and the GST tag was removed on-column with 100 U/ml thrombin (Sigma-Aldrich) at 4°C and eluted with GF500 buffer. The eluted proteins were subsequently subjected to a HiLoad 16/600 Superdex 200 Prep Grade column (GE Healthcare) using a semi-automated ÄKTA Purifier FPLC purification system (GE Healthcare) in filtered GF150 buffer containing 20 mM HEPES at pH 7.4 and 150 mM NaCl (for ITC experiments) or GF500 buffer with the addition of 2 mM dithiothreitol (DTT; for crystallisation).

### Generation of nanobodies

An alpaca was immunised six times with 200 μg of purified recombinant mC1-HR1. The adjuvant used was GERBU FAMA. Immunisation and handling of the alpacas for scientific purposes was approved by Agriculture Victoria, Wildlife & Small Institutions Animal Ethics Committee, project approval no. 26-17. Blood was collected 3 days after the last immunisation for the preparation of lymphocytes. Nanobody library construction was carried out according to established methods as described previously (Dietrich et al., 2021). Briefly, alpaca lymphocyte mRNA was extracted and amplified by reverse transcription PCR with specific primers to generate a cDNA nanobody library. The library was cloned into a pMES4 phagemid vector (NovaPro, V004765), amplified in *E. coli* TG1 strain and subsequently infected with M13K07 helper phage (ThermoFisher, 18311019) for recombinant phage expression.

Biopanning for recombinant mC1-HR1 nanobodies using phage display was performed as previously described with following modifications (Dietrich et al., 2021). Phages displaying mC1-HR1-specific nanobodies were enriched after two rounds of biopanning on immobilised mC1-HR1. After the second round of panning, we screened single clones by ELISA (Dietrich et al., 2021), and positive clones were selected for further analysis. 43 positive clones were sequenced and seven distinct nanobody clonal groups were identified based on unique CDR3 sequences.

### Recombinant protein expression and purification of nanobodies

Nanobodies were expressed in *E. coli* WK6 cells and purified as previously described (Dietrich et al., 2021). Nanobodies were cloned with a C-terminal 6× histidine tag into pMES4 expression vector for periplasmic expression in *E. coli* WK6 (*Su*^−^) (Zell and Fritz, 1987). Nanobodies were expressed in Terrific Broth medium (ThermoFisher, 22711022) supplemented with 100 mg/ml ampicillin, 20% glucose and 2 mM MgCl_2_, and then induced with 1 mM IPTG at 25°C for 20 h. To extract proteins from the periplasm by osmotic shock, the harvested cell pellets were resuspended in 200 ml TES buffer containing 0.2 M Tris-HCl at pH 8.0, 0.5 mM EDTA and 0.5 M sucrose with 50 mg/ml benzamidine hydrochloride, and incubated on an orbital shaking platform at 4°C overnight. 400 ml of 0.25× concentration TES buffer was then added to the resuspended pellet and shaken for another 1 h at 4°C. The lysates were centrifuged at 14,000 ***g*** for 30 min to extract proteins. His-tagged nanobodies were purified by TALON metal affinity resin and then subjected to size-exclusion chromatography in a HiLoad 16/600 Superdex 200 Prep Grade column using a semi-automated ÄKTA Purifier FPLC purification system.

### GST pulldown assay

GST pulldown assay was carried out using the GST-tagged mC1-HR1 domain as the bait protein for His-tagged Cavin1-specific nanobodies NbA4, NbA12, NbB7, NbD5, NbD6, NbH3 and NbH5. 1 nmol of GST-tagged mC1-HR1 and GST alone were mixed with 2 nmol of His-tagged Cavin1-selected nanobodies in 500 µl of pulldown buffer (20 mM HEPES, 150 mM NaCl, 1 mM DTT, 0.1% IGEPAL, pH 7.4). The protein mixture was incubated on a shaking platform for 1 h at 4°C. Then, the mixture was centrifuged at 5000 rpm in a Thermo Fisher Pico17 bench centrifuge for 20 min to remove any precipitated proteins. The clarified protein mixture and GST alone were incubated with 50 μl of pre-equilibrated glutathione Sepharose affinity resin (GE healthcare). The reaction mixtures were incubated at 4°C for 1 h and the protein-bound glutathione Sepharose affinity resin was spun down at 2000 ***g*** for 30 s. After removing the supernatant, the resin was washed four times with pulldown buffer. The resin was then mixed with 50 μl of SDS sample buffer and analysed by SDS-PAGE.

### ITC

All microcalorimetry experiments were conducted in GF150 buffer at 25°C using a MicroCal PEAQ-ITC system (Malvern). ITC experiments were performed with a single 0.4 μl injection followed by 12 injections of 3.22 μl each with 180 s injection spacing. NbA12 at 500 μM and NbB7 at 400 μM were titrated into 30 μM of the monomeric HR1 domain of cavins and His-MBP full-length mC1, respectively. The interaction of the mC1-HR1 domain and NbB7 in the presence of the competing nanobody NbA12 was conducted by titrating 1 mM NbB7 into 50 μM mC1-HR1 pre-incubated with 50 μM NbA12. 200 μM NbA12 or NbB7 was titrated into 6.25 μM trimeric mC1-HR1 HT/II or TS/DD mutant. Data were analysed by Malvern software by fitting and normalised to a single-site-binding stoichiometry, and thermodynamic profiles were presented using Prism v8.0.1 (GraphPad Software). Experiments were conducted with at least three technical replicates for data reproducibility.

### Crystallisation and data collection

The hanging-drop vapor diffusion method was applied for crystallisation screening under a 96-well plate using a Mosquito Liquid Handling robot with a 1:1 ratio of protein and reservoir solution at 20°C. To co-crystallise nanobodies with the mC1-HR1 domain, a 1.5-fold molar excess of purified nanobody NbA12 or NbB7 was incubated separately to the purified mC1-HR1 domain, followed by size-exclusion chromatography to form a complex with a final concentration of 12.6 mg/ml. Initial crystals of the NbB7–mC1-HR1 complex were obtained in many commercial screen conditions, but the best diffraction-quality crystal was optimised in 0.65 M potassium thiocyanate and 19.5% PEG 2000 MME.

For co-crystallisation of mC1-HR1 HT/II and TS/DD mutants with NbB7, the GST tag on the HR1 mutants was cleaved by thrombin and pre-incubated with a 1.5-fold molar excess affinity-purified NbB7 overnight to form a complex. The protein complex crystals were produced using the hanging-drop method in the crystallisation condition containing 0.2 M potassium thiocyanate, 20% PEG 3350 and 0.2 M potassium citrate, 20% PEG 3350, respectively.

All the crystals were soaked in the appropriate cryoprotectant solutions containing the crystallisation solutions supplemented with 25% glycerol, and flash-cooled in a nitrogen gas stream for transport to the Australian Synchrotron for data collection. X-ray diffraction data were measured on the MX2 beamline at the Australian Synchrotron.

### Crystal structure determination

The collected data were integrated using XDS (Kabsch, 2010) and scaled with AIMLESS (Evans and Murshudov, 2013). The crystal structures of NbB7 in complex with mC1-HR1 and the HT/II and TS/DD mutants were solved by molecular replacement using PHASER (McCoy et al., 2007), with the available mC1-HR1 domain [Protein Data Bank (PDB) ID: 4QKV] and GFP nanobody (PDB ID: 3OGO) structures as the initial models. The initial electron density map was then refined in PHENIX suite (Adams et al., 2010) and rebuilt in COOT (Emsley et al., 2010). Data and refinement statistics are summarised in Table S2 and molecular figures are presented using PyMOL.

### Liposome preparation and pelleting assay

Folch liposomes were freshly prepared using 100% Folch fraction I (Sigma-Aldrich) in chloroform to generate a lipid film in a mini round-bottomed flask and dried gently under a nitrogen stream and then under vacuum overnight. To form multilamellar vesicles, the dried lipid film was rehydrated in GF150 buffer, followed by ten rapid freeze-thaw cycles using acetone in dry ice and 60°C water. The pelleting assay was conducted in a total volume of 100 μl comprising 50 μl of 1 mg/ml multilamellar vesicles and 50 μl of 10 μM GST–mC1-HR1, 10 μM NbA12 or NbB7 and 10 μM GST–mC1-HR1 in complex with NbA12 or NbB7 in a 1:1 ratio. The reaction mixtures were incubated at 25°C for 10 min followed by centrifugation at 60,000 ***g*** for 15 min at 4°C in an Optima MAX-XP tabletop ultracentrifuge (Beckman Coulter) using a TLA100 rotor. The supernatant was collected, and the pellet was carefully separated from the supernatant and resuspended in 50 μl GF150 buffer containing 4× loading dye, before analysis by SDS-PAGE.

### Liposome preparation and tubulation assay

The liposome tubulation assay was perfumed as described previously (Kovtun et al., 2014). Liposomes were prepared as described above and rehydrated in GF150 buffer followed by three repetitive freeze-thaw cycles to form multilamellar layers. Large unilamellar lipid vesicles were generated by subjecting the liposomes to 21 rounds of extrusion through an 800-nm polycarbonate membrane (Avanti, 610005) using an Avanti miniextruder. Purified complexes of NbA12 or NbB7 pre-incubated with His-MBP full-length mC1 at 0.1 mg/ml were incubated with 0.2 mg/ml liposomes for 2 min at 25°C. Samples were then immediately applied onto formvar carbon-coated electron microscopy grids (300 mesh) for 10 s. Excess samples were eliminated by blotting with the corners of Whatman filter paper. The grids were washed with distilled water three times before application of 2% uranyl acetate stain. The grids were blotted again to remove excess stain and left to air dry before viewing. Images were captured using a JEOL 1011 transmission electron microscope at 80 kV.

### Cell culture and transfection

Cell lines were sourced from the American Type Culture Collection (ATCC). HeLa, A431 and BHK cells were maintained in Dulbecco's modified Eagle medium (Thermo Fisher Scientific) supplemented with 10% fetal bovine serum (Scientifix, FBSFR-S00JF) and 2 mM L-glutamine (Thermo Fisher Scientific) at 37°C. PC3 cells were maintained in RPMI medium (Gibco, 72400047). On the following day, HeLa, A431, PC3 and BHK cells were transfected with Lipofectamine 2000 (Themo Fisher Scientific) at 70% confluency. Cavin1 knockout HeLa cells (Wu et al., 2023) were transfected with Lipofectamine 3000 (Thermo Fisher Scientific) at 70% confluency according to the manufacturer's instructions.

### Co-immunoprecipitation

NHS-activated Sepharose 4 fast flow (Cytiva, GEHE17-0906-01) resin was prepared by washing with GF500 buffer and coupled with a GFP-tagged nanobody (NbA12 or NbB7) with overnight rotation according to the manufacturer's protocol. The unbound GFP nanobody was removed by washing with GF500 buffer and the resin blocked by 0.2 M ethanol amide for 5 h. The coupled resin was washed two times with GF500 buffer before storage in PBS at 4°C.

HeLa cells were cultured on Corning tissue-culture dishes until they reached 50–70% confluency. Cells were removed by mechanical scraping from dishes and harvested by centrifugation at 500 ***g*** for 10 min. The harvested cells were lysed in ice-cold GF150 buffer with 0.5% Triton X-100, 0.1 mg/ml benzamidine, 10 μg/mL DNase I, phosphatase inhibitor cocktail (Roche, PhosSTOP) and protease inhibitor cocktail (cOmplete, EDTA-free). They were lysed by sonication using an ultrasonic cell disruptor with three pulses at a time on ice and centrifuged at 1000 ***g*** for 2 min. The supernatant was collected from cell lysates and 100 μl of supernatant was boiled with 40 μl 4× SDS-PAGE buffer as lysate control for analysis. The rest of the supernatant was subjected to a 2 h incubation at 4°C with NbA12/NbB7-coupled NHS-activated Sepharose resin. The resultant pellet was then washed with ice-cold GF150 buffer three times and then suspended in 200 μl of GF150 buffer with 60 μl 4× SDS-PAGE buffer for western blot analysis.

Whole-cell lysates were collected from HeLa cells grown in the six-well dishes and lysed with RIPA buffer (150 mM NaCl, 0.5% Triton X-100, 0.1% SDS, 50 mM Tris, pH 7.4), supplemented with phosphatase inhibitor cocktail (Roche, PhosSTOP) and protease inhibitor cocktail (cOmplete, EDTA-free) at 4°C. After scratching from the bottom of six-well plates and vortexing for homogenisation, the cell lysates were incubated on ice for 5 min and centrifuged at 10,000 ***g*** for 3 min at 4°C. The supernatant was collected and boiled with 4× SDS-PAGE buffer at 95°C for 3 min for sample collection.

### Western blotting

Samples were separated on SDS-PAGE gel in 1× MES buffer at 90 V for the first 20 min and 130 V until the dye front reached the bottom of the gel. The proteins were transferred from SDS-PAGE gels to polyvinylidene difluoride (PVDF) membranes via dry transfer according to the western blotting protocol by Abcam (https://docs.abcam.com/pdf/protocols/general-western-blot-protocol.pdf). The PVDF membrane was blocked by incubation with 5% skim milk in PBS containing 0.1% Tween-20 (PBST) for 1 h at room temperature to prevent non-specific binding. The PVDF membranes were then incubated with anti-GFP (polyclonal mouse, Proteintech, 50430-2-AP, 1:1000), anti-Cavin1 (polyclonal rabbit, Proteintech, 18892-1-AP, 1:1000) or anti-CAV1 (polyclonal rabbit, BD Transduction Laboratories, 610059, 1:1000) primary antibodies in 5% skim milk in PBST overnight at 4°C. The membrane was washed four times with PBST for 5 min and then incubated with appropriate secondary antibodies (1:2000 for horseradish peroxidase-conjugated goat anti-mouse IgG, BioRad, 1706516; 1:12,000 for horseradish peroxidase-conjugated goat anti-rabbit IgG, BioRad, 1706515) in PBST with 5% skim milk for 90 min. The membrane was washed again with PBST three times and developed using chemiluminescence-based immunodetection by Novex chemiluminescent substrate (Invitrogen). Images were then obtained by Hi-resolution blot imaging (BioRad Chemidoc MP Imaging System) and normal image scanning for colorimetric detection following the manufacturer's instructions.

### Immunofluorescence imaging and confocal microscopy

Cells expressing GFP nanobody NbB7 or NbA12 constructs were plated on glass coverslips and fixed with 4% paraformaldehyde in PBS for 30 min at room temperature. They were then washed with PBS and permeabilised with 0.1% Triton-X 100 in PBS, followed by the addition of 2% bovine serum albumin for 1 h for blocking. Cells were probed with the primary antibody anti-Cavin1 (polyclonal rabbit, Proteintech, 50430-2-AP, 1:800) or anti-CAV1 (polyclonal rabbit, BD Transduction Laboratories, 610059, 1:800) for 45 min at 4°C. Secondary antibodies (goat anti-rabbit IgG, BioRad, 1706515, dilution 1:400) were then incubated on coverslips for 45 min at room temperature. The coverslips were mounted in Mowiol in PBS. Images were acquired using an inverted LSM 880 Fast Airyscan microscope (Zeiss) equipped with 63× oil immersion objective lens.

Cavin1 knockout HeLa cells were seeded onto glass coverslips and fixed with 4% paraformaldehyde in PBS for 15 min at room temperature. Following fixation, cells were washed three times with PBS. Coverslips were then mounted in Mowiol in 0.2 M Tris-HCl, pH 8.5. Images were captured using a Zeiss LSM 880 confocal microscope. Intensity line-scan analysis was conducted in ImageJ 2.0; the ‘line’ tool was used to draw a line at the measurement site in the first channel and repeated in the second channel, and fluorescence intensities were measured across the line in both channels using the ‘plot profile’ option.

### APEX labelling electron microscopy

BHK cells were seeded into 35-mm tissue culture dishes overnight and then transfected with Lipofectamine 3000 as per the manufacturer's instructions. Dishes were fixed 24 h later with 2.5% glutaraldehyde in PBS and then washed repeatedly in PBS. Fixed cells were treated with a freshly prepared solution of 0.05% 3,3′-diaminobenzidine (DAB, Sigma-Aldrich) in PBS for 10 min, followed by incubation in a solution of 0.05% DAB containing 0.01% H_2_O_2_ for 30 min at room temperature. Cells were postfixed with 1% osmium for 2 min and underwent a series of dehydration steps using increasing percentages of ethanol. Cells were then subjected to a series of infiltration steps with LX112 resin (Ladd Research, 21310) in a Pelco Biowave system, followed by 24 h incubation at 60°C for polymerization. Ultrathin sections were attained on a ultramicrotome (UC6, Leica) and imaged using a JEOL1011 transmission electron microscope at 80 kV.

### Differential scanning fluorimetry

Thermal unfolding assay was conducted using a ViiA7 real-time PCR instrument (Applied Biosystems) to detect preferential binding of a fluorophore to unfolded molecules. Briefly, 2 mg/ml of freshly purified mC1-HR1 (control) and HT/II and TS/DD mutants were centrifuged at 15,000 ***g*** at 4°C for 15 min to eliminate possible precipitants and then mixed with freshly prepared 500× SYPRO orange dye (Life Sciences) to a final concentration of 8 μM for thermal denaturation measurement. The sample mixture was then loaded onto a 96-well plate and experiments were performed in four replicates. Relative fluorescence units were measured from 24 to 80°C with 1°C increments using ROX dye calibration (ThermoFisher, 12223012). The melting temperature (T_m_) was determined by fitting the data into a Boltzmann sigmoidal curve in Prism v10.2.0 (GraphPad Software).

## Supplementary Material



10.1242/joces.263756_sup1 Supplementary information
