# Supplementary material for: Nanobodies against Cavin1 reveal structural flexibility and regulated interactions of its N-terminal coiled-coil domain
Source: J Cell Sci. 2025 Apr 28;138(8):jcs263756. doi: 10.1242/jcs.263756 (PMC12079668; doi:10.1242/jcs.263756)
Supplement: Supplementary information [file joces-138-263756-s1.pdf]

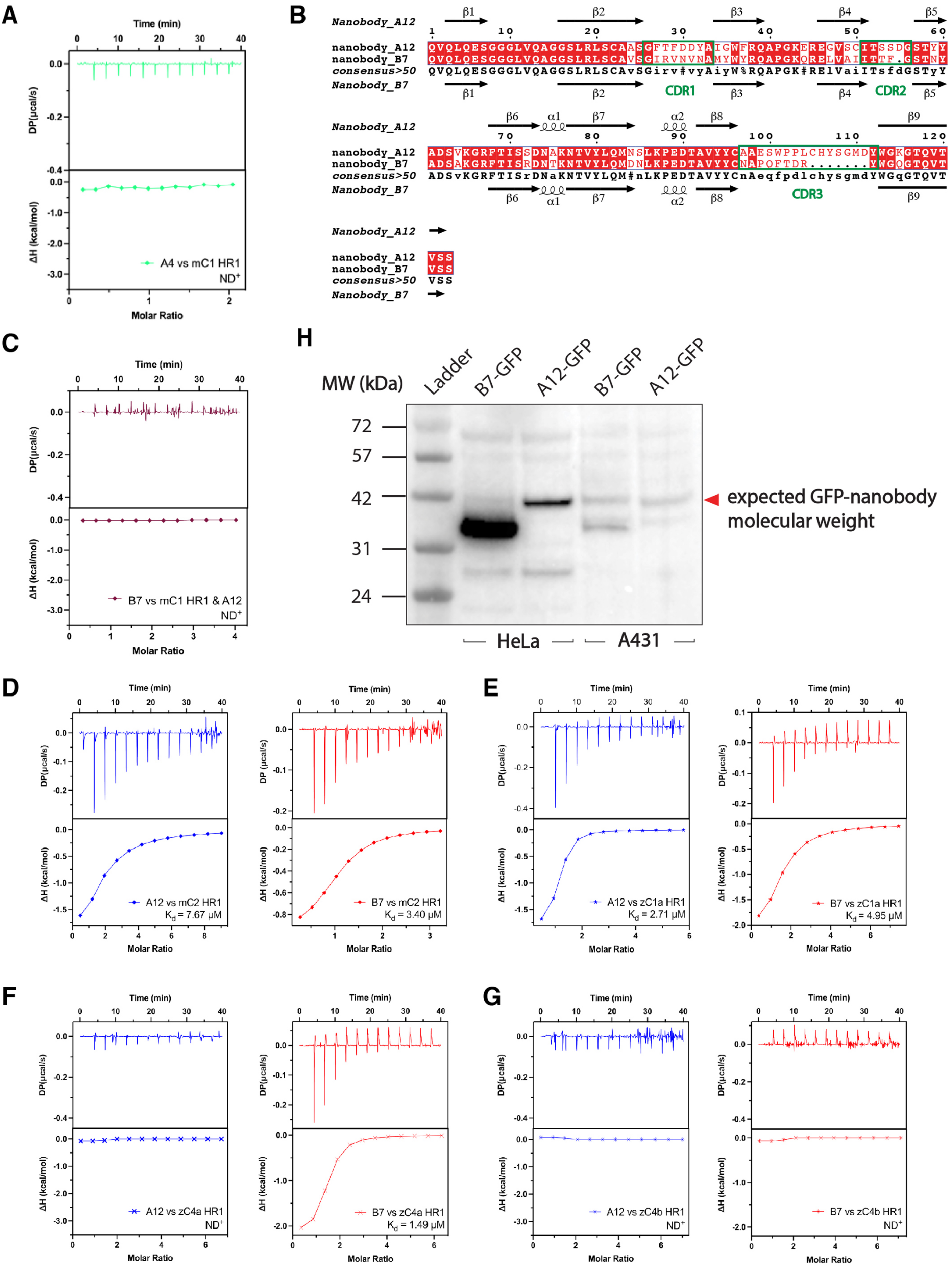

Journal of Cell Science • Supplementary information

Fig. S1. Nanobody sequences, binding *in vitro*, and expression in cells.
(A) ITC experiment showing lack of affinity between NbA4 and mC1-HR1. (**B**) Sequence alignment of nanobody NbA12 and NbB7. The three CDRs are highlighted in green boxes. **(C)** Nanobody NbA12 and NbB7 competes for binding with mouse Cavin1 HR1. ND^{+}: no binding detected. **(D)** Binding of NbA12 and NbB7 with mouse Cavin2 HR1 domain measured by ITC. **(E)** Binding of both nanobodies with zebrafish Cavin1a HR1 domain measured by ITC. **(F)** Binding of nanobody NbB7 with zebrafish Cavin4a HR1 domain was measured by ITC but no binding was observed for nanobody NbA12. **(G)** ITC thermograms showing that both nanobodies could not interact with zebrafish Cavin4b HR1 domain. ND^{+}: no binding detected. The upper panel represents raw data, and the lower panel represents the normalized and integrated binding isotherms fitted with a 1-to-1 binding ratio. The binding affinity (K_d) is determined by calculating the mean of at least two independent experiments. (**H**) Western blot with anti-GFP antibody to detect expression of NbB7-GFP and NbA12-GFP in HeLa cells and A431 cells. Expression levels are higher in HeLa cells, and suggest that some nicking of the NbB7 protein occurs at higher expression levels.

Journal of Cell Science • Supplementary information

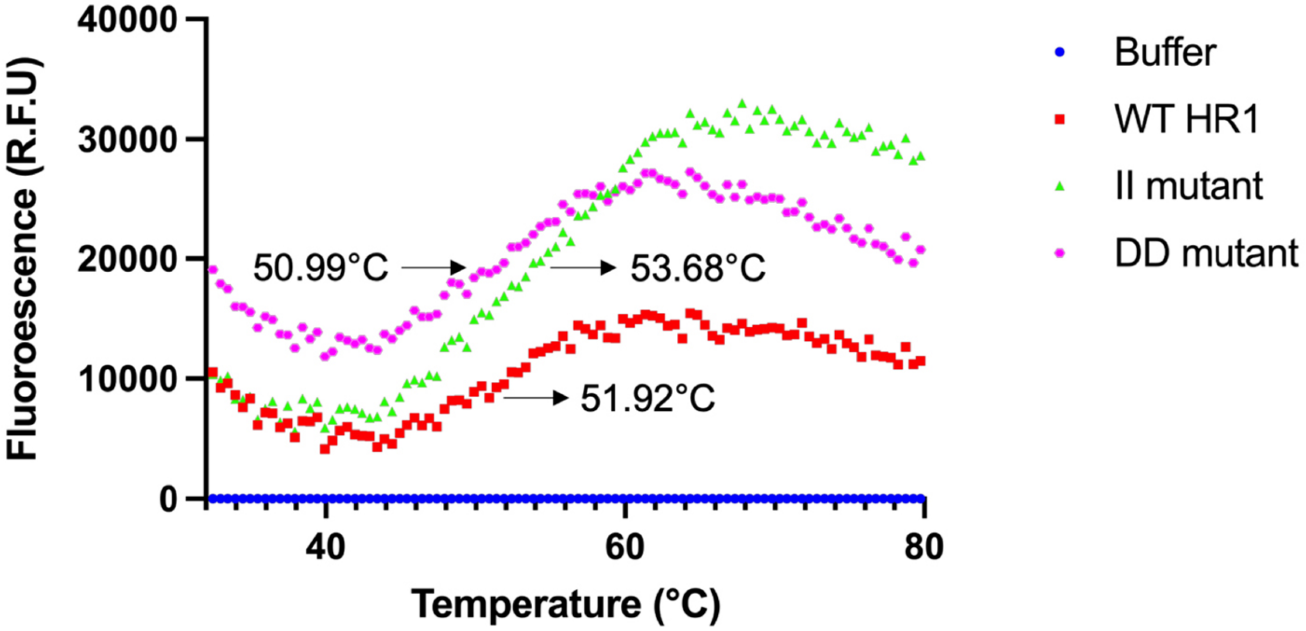

Fig. S2. Thermal stability of Cavin1 HR1 domain mutants.

(A) Differential scanning fluorimetry (DSF) assay to measure the melting temperature of the mC1-HR1 trimer and comparison with the HT/II and TS/DD mutants.

Journal of Cell Science • Supplementary information

Table S1. Thermodynamic parameters for the interaction of cavin proteins with nanobodies by ITC

	K_d (µM)	ΔH (kcal/mol)	ΔG (kcal/mol)	-TΔS (kcal/mol)
**NanobodyNbA12**				
mC1 HR1	0.50 ± 0.14	-5.70 ± 0.08	-8.57 ± 0.16	-2.88 ± 0.23
mC1 HR1 45-103	0.57 ± 0.00	-5.07 ± 0.09	-8.54 ± 0.01	-3.35 ± 0.07
mC1 HR1 5Q	0.14 ± 0.02	-4.09 ± 0.83	-9.35 ± 0.11	-5.26 ± 0.73
mC1 full-length	8.88 ± 1.73	-0.48 ± 0.09	-6.90 ± 0.11	-6.42 ± 0.21
mC2 HR1	7.67 ± 6.55	-1.74 ± 0.23	-7.12 ± 0.59	-5.38 ± 0.82
zC1a HR1	2.71 ± 2.38	-2.75 ± 1.25	-7.75 ± 0.62	-5.00 ± 1.86
zC4a HR1	No binding detected			
zC4b HR1	No binding detected			
**Nanobody NbB7**				
mC1 HR1	0.52 ± 0.35	-4.21 ± 0.49	-8.67 ± 0.45	-4.45 ± 0.93
mC1 45-103	0.45 ± 0.14	-4.77 ± 0.52	-8.68 ± 0.19	-3.91 ± 0.71
mC1 HR1 5Q	0.89 ± 0.02	- 4.45 ± 1.09	-8.26 ± 0.01	-3.81 ± 1.11
mC1 full-length	1.73 ± 0.18	-6.39 ± 0.52	-7.87 ± 0.06	-1.48 ± 0.58
mC2 HR1	3.40 ± 2.46	-0.78 ± 0.09	-7.56 ± 0.47	-6.77 ± 0.38
zC1a HR1	4.95 ± 2.47	-2.01 ± 1.46	-7.28 ± 0.31	-5.28 ± 1.76
zC4a HR1	1.64 ± 0.21	-2.26 ± 0.05	-7.90 ± 0.07	-5.65 ± 0.02
zC4b HR1	No binding detected			
**NbB7 vs mC1 HR1 & NbA12**	No binding detected			

Journal of Cell Science • Supplementary information

Table S2. Statistics summary of X-ray crystallographic structure determination

	**Nanobody NbB7-mouse Cavin1 HR1**	**Nanobody NbB7-mouse Cavin1 HR1 HT/II**	**Nanobody NbB7-mouse Cavin1 HR1 TS/DD**
**Data collection statistics**			
PDB ID	9EGN	9EG6	9EIU
Space group	P63	C 1 2 1	P 3
Resolution (Å)	46.50 – 1.57	48.82 – 3.2	30.6-4.0
a, b, c (Å)	58.90, 58.90, 113.12	102.32, 59.24, 133.82	58.67, 58.67, 61.29
α, β, γ (°)	90.0, 90.0, 120.0	90.0, 97.48, 90.0	90.0, 90.0, 120.0
Total observations	641590 (27806)	87884 (15457)	10543 (3143)
Unique reflections	31084 (1492)	13349 (2402)	1975 (572)
Completeness (%)	99.7 (95.0)	99.8 (100.0)	99.8 (99.8)
R_{merge}^{+}	0.045 (0.469)	0.297 (1.281)	0.076 (0.159)
R_{pim*}	0.010 (0.107)	0.116 (0.502)	0.037 (0.077)
CC1/2	1.000 (0.958)	0.991 (0.878)	0.998 (0.990)
$<I/\sigma(I)>$	28.3 (4.0)	6.6 (2.6)	7.9 (6.3)
Multiplicity	20.6 (18.6)	6.6 (6.4)	5.3 (5.5)
Wilson B (Å^2)	21.85	59.30	113.89
**Refinement statistics**			
Reflections used in refinement	31044 (2149)	13281 (2634)	1976 (1976)
Reflections used for R-free	2001 (140)	721 (154)	130 (130)
R-work	0.1727 (0.2158)	0.2584 (0.3596)	0.2022 (0.2022)
R-free	0.1904 (0.2400)	0.2870 (0.4261)	0.2618 (0.2618)
Number of non-hydrogen atoms	1351	3597	1211
macromolecules	1211	3597	1211
ligands	0	0	0
solvent	140	0	0
Protein residues	157	466	157
RMS(bonds) (Å)	0.008	0.005	0.003
RMS(angles) (°)	1.11	0.76	0.71
Ramachandran favored (%)	98.69	98.68	98.69
Ramachandran allowed (%)	1.31	1.32	1.31
Ramachandran outliers (%)	0.00	0.00	0.00
Rotamer outliers (%)	0.00	0.00	0.00
Clashscore	1.24	2.24	0.83
Average B-factor (Å^2)^	28.87	105.15	183.05
macromolecules	27.58	105.15	183.05
solvent	40.01	-	

*Values in parentheses refer to the highest resolution shell. +Rmerge = Σ|I – <I> |⁄Σ<I>, where I is the intensity of each individual reflection. *Rpim indicates all I+ & I-. ¶Rwork = Σh|Fo - Fc|/ ΣI| Fo|, where Fo and Fc are the observed and calculated structure-factor amplitudes for each reflection h. Rfree# was determined by randomly selecting 10% of the diffraction data and excluded from refinement. Average B (Å2)^ was calculated using Baverage.*

Journal of Cell Science • Supplementary information

Table S3. Thermodynamic parameters for the interaction of mouse Cavin1 HR1 mutants with nanobodies by ITC

Nanobody NbB7	K_d (µM)	ΔH (kcal/mol)	ΔG (kcal/mol)	-TΔS (kcal/mol)
mC1 HR1 Q69A	No binding detected			
mC1 HR1 L72E	No binding detected			
mC1 HR1 Q76A	2.89 ± 1.01	-1.02 ± 0.34	-7.58 ± 0.21	-6.55 ± 0.13

Table S4. Thermodynamic parameters for the interaction of mouse Cavin1 HR1 HT/II and TS/DD mutants with nanobodies by ITC

	K_d (µM)	ΔH (kcal/mol)	ΔG (kcal/mol)	-TΔS (kcal/mol)
**Nanobody NbB7**				
mC1 HR1 HT/II mutant	0.14 ± 0.04	-4.46 ± 0.66	-9.36 ± 0.16	-4.90 ± 0.82
mC1 HR1 TS/DD mutant	0.067 ± 0.02	-5.44 ± 0.37	-9.82 ± 0.22	-4.37 ± 0.58
**Nanobody NbA12**				
mC1 HR1 HT/II mutant	3.02 ± 1.94	-1.34 ± 0.49	-7.60 ± 0.41	-6.27 ± 0.08
mC1 HR1 TS/DD mutant	2.17 ± 0.02	-1.11 ± 0.33	-7.73 ± 0.01	-6.62 ± 0.33

Journal of Cell Science • Supplementary information

Table S5. DNA constructs of cavin proteins and nanobodies

Species	Construct	Affinity tag	Antibiotics	Vector
Mouse	Cavin1 HR1 (residues 45-155)	His-Ub	Amp	pHUE
Mouse	Cavin1 HR1 (residues 45-103)	His-Ub	Amp	pHUE
Mouse	Cavin1 HR1 5Q (residues 45-155)	His-Ub	Amp	pHUE
Mouse	Cavin2 HR1 (residues 47-157)	His-Ub	Amp	pHUE
Zebrafish	Cavin1a HR1 (residues 73-193)	His-Ub	Amp, Cam	pHUE
Zebrafish	Cavin4a HR1 (residues 16-123)	His-Ub	Amp, Cam	pHUE
Zebrafish	Cavin4b HR1 (residues 12-133)	His-Ub	Amp, Cam	pHUE
Mouse	Cavin1 HR1 Q69A (residues 45-155)	His-Ub	Amp	pHUE
Mouse	Cavin1 HR1 L72E (residues 45-155)	His-Ub	Amp	pHUE
Mouse	Cavin1 HR1 Q76A (residues 45-155)	His-Ub	Amp	pHUE
Mouse	Cavin1 full-length (residues 1-392)	His-MBP	Amp	2K-T
Mouse	Cavin1 HR1 (residues 45-155)	GST	Kan, Cam	pGEX-4T2
Mouse	Cavin1 HR1 I102-I105 (HT/II)	GST	Kan, Cam	pGEX-4T2
Mouse	Cavin1 HR1 D105-D106 (TS/DD)	GST	Kan, Cam	pGEX-4T2
Mouse	Cavin1 Q69A (residues 1-392)	GFP	Kan	pEGFP-N1
Mouse	Cavin1 L72E (residues 1-392)	GFP	Kan	pEGFP-N1
Mouse	Cavin1 Q76A (residues 1-392)	GFP	Kan	pEGFP-N1
Alpaca	Nanobody NbA12	GFP	Kan	pEGFP-N1
Alpaca	Nanobody NbB7	GFP	Kan	pEGFP-N1
Mouse	Cavin1 HT/II	GFP	Kan	pEGFP-N1
Mouse	Cavin1 TS/DD	GFP	Kan	pEGFP-N1

**Amp-Ampicillin; Kan-Kanamycin; Cam-chloramphenicol.*

Table S6. Key resources

REAGENT OR RESOURCE	SOURCE OR REFERENCE	IDENTIFIER
**Bacterial Strains**		
E. coli DH5α	Invitrogen	18265017
E. coli BL21 (DE3)	Invitrogen	C600003
E. coli BL21 CodonPlus™ (DE3)	Agilent Technologies	230245
**Chemicals**		
Isopropyl ß-D-1-thiogalactopyranoside	Bioline	BIO-37036
Benzamidine hydrochloride hydrate	Sigma Aldrich	B6506
Deoxyribonuclease I (DNase I)	Sigma Aldrich	DN25
Imidazole	Sigma Aldrich	792527
TALON® resin	Clontech	635503
Glutathione Sepharose 4B	GE Healthcare	GEHE17-0756-0
Folch fraction I	Sigma Aldrich	B1502
**Software**		
XDS	(Kabsch, 2010)	http://xds.mpimf-heidelberg.mpg.de/
AIMLESS	(Evans and Murshudov, 2013)	http://www.ccp4.ac.uk/html/aimless.html
Phaser	(McCoy et al., 2007)	http://www.phaser.cimr.cam.ac.uk/index.php/Phaser_Crystallographic_Software
Phenix	(Adams et al., 2010)	https://www.phenix-online.org/
Coot	(Emsley and Cowtan, 2004)	https://www2.mrc-lmb.cam.ac.uk/personal/pemsley/coot/
Pymol	(Schrodinger, USA)	https://pymol.org/2/
**Other items**		
HiLoad™ 16/600 Superdex™ 200	GE Healthcare	10223894

Journal of Cell Science • Supplementary information
