# Supplementary material for: Nanobodies against Cavin1 reveal structural flexibility and regulated interactions of its N-terminal coiled-coil domain
Source: J Cell Sci. 2025 Apr 28;138(8):jcs263756. doi: 10.1242/jcs.263756 (PMC12079668; doi:10.1242/jcs.263756)
Supplement: Supplementary file 1 [file joces-138-263756_review_history.pdf]

Nanobodies against Cavin1 reveal structural flexibility and regulated interactions of its N-terminal coiled-coil domain

Ya Gao, Vikas A. Tillu, Yeping Wu, James Rae, Thomas E. Hall, Kai-En Chen, Saroja Weeratunga, Qian Guo, Emma Livingstone, Wai-Hong Tham, Robert G. Parton and Brett M. Collins
DOI: 10.1242/jcs.263756

Editor: Michael Way

Review timeline
Original submission: 27 November 2024
Editorial decision: 7 January 2025
First revision received: 4 March 2025
Accepted: 6 March 2025

Original submission

First decision letter

MS ID#: jcs.263756

MS TITLE: Cavin1 binding nanobodies reveal structural flexibility and regulated interactions of the N-terminal coiled-coil domain

AUTHORS: Ya Gao; Vikas Tillu; Yeping Wu; James Rae; Thomas Hall; Kai-En Chen; Saroja Weeratunga; Qian Guo; Emma Livingstone; Wai-Hong Tham; Robert Parton; Brett Collins

ARTICLE TYPE: Research Article

Dear Dr Collins,

We have now reached a decision on the above manuscript.

To see the reviewers' reports and a copy of this decision letter, please go to:

As you will see, the reviewers raise a number of criticisms that prevent me from accepting the paper at this stage. They suggest, however, that a revised version might prove acceptable, if you can address their concerns. If you think that you can deal satisfactorily with the criticisms on revision, I would be pleased to see a revised manuscript.

Reviewer 1

Advance summary and potential significance to field

In this manuscript, the authors, Gao et al., use nanobodies to characterize the structure of the helical region (HR1) of cavin1 in solution and bound to membranes and caveolae. They describe the development and characterization of these nanobodies, which could be very useful to the field. The study uses a combination of protein crystallization, biochemical analysis of reconstituted systems and microscopy analysis of proteins on caveolae in cells. They show that the helical bundling of the C-terminal region of the trimeric HR1 is relatively instable. Using the specificity of different nanobodies, they report that the C-terminal region does not form a helical coiled coil when associated with caveolae in agreement with previous modelling work. The experiments are sound

© 2025. Published by The Company of Biologists under the terms of the Creative Commons Attribution License (https://creativecommons.org/licenses/by/4.0/).

and the conclusions are supported by the data. This is very interesting and important work that advance the caveolae field but also contributes to our structural understanding peripherally attached membrane proteins.

Comments for the author

Suggestions for improvements:

1. The manuscript would majorly benefit from overall improvement of the representation of the data, figure outline and labelling. Check labeling, scale bar colors etc of all figures. Examples:
- It would be more intuitive to the reader if the ITC for A12 and B7 were kept to one color each throughout the paper.
- Figure 1F is referenced in the main text as 1G.
- In the figure legend figure 1B is referenced as 1C and vice versa.
- Fig 4A; Confusing labeling of input and Co-ip. In the figure legend it is stated that western blot was performed with antibodies against Cavin1 and Caveolin1, antibody against GFP should be included.
- Fig 4 B-C; If possible, remove text from the scale bar in the images.
- Fig 4B; In the main text it is stated that colocalization of the nanobodies and CAV1 in HeLa cells was tested but no such image exists within the manuscript.
- Fig 4C; Why not show the colocalization of Cavin1 and the nanobodies as in B? Is there any particular reason for using the A431 cells, please specify in the main text.
- Fig 4D; Are there any visible caveolae in the NbA12 image? Why is this image in a dashed line box? Make the figure cohesive with the NbB7 image. It is not clear from the legend which size the different scale bars have.
- Fig 6; Remove the zoom out images (top images). Make the presentation of the profile analysis cohesive in terms of ticks on the Y-axis.
- Fig 8; Different sizes of the images are shown. Make A and B as in C and D.

2. The data in Figures 1-3 is about the characterization of the nanobodies and in my view, not all the ITC data needs to be in the main figures. If some of the data are moved to supplementary data, then I think it would be possible to have just one figure for the characterization of the nanobodies. In this way more focus will be put on the conclusions drawn by using the nanobodies.

3. In Fig 1A the A4 nanobody is claimed to have affinity for GST-HR1. Yet, since the amount of A4 nanobody is much higher in also the supernatant, I doubt that this is a relevant signal. By instead evaluating the ratio of the pellet and total (pellet plus supernatant) for each nanobody, I find it more likely that it is only A12 and B7 that show significant binding. This would also be much more consistent with the ITC data following up on these nanobodies.

4. The authors have tested different Cavin1 mutants and state in the abstract that the conserved buried hydrogen-bonded His-Thr pair mutation reveals a selective regulation of Cavin2/Cavin3 association to Cavin1. This should be corrected in the abstract since they show that this mutation does not affect the localization of either Cavin2 or Cavin3. They do however show that it is the phosphomimic TS/DD mutation that impact the localization of Cavin2 to caveolae.

5. In the results section the choice of cell lines for different experiments could be presented to the reader.

6. I don't understand the statement starting on line 169 regarding Fig S1D. Lower expression levels of what? Specify if the NbB7-GFP is expressed in higher levels.

7. Reformulate statement line 284 "Caveolae formation and recruitment …."
By expression of only cavin1, cavin2 and cavin 3 cannot be rectruited if they are not expressed in the cells?

8. I don't understand what is meant by "plasma" on line 224 and 225? Plasma membrane?

9. I would suggest to remove the sentence starting on line 278 since it is not required

© 2025. Published by The Company of Biologists under the terms of the Creative Commons Attribution License (https://creativecommons.org/licenses/by/4.0/).

Reviewer 2

Advance summary and potential significance to field

This paper characterizes the nanobody binding to the Cavin1 HR1 domain. The nanobody binding would induce the unfolding of the part of Cavin1. The mutants of Cavin1 also exhibited an unfolded structure. The nanobody had a higher affinity to these mutants, suggesting the preference for the unfolded Cavin, which would reflect the differential staining by the antibodies in comparison with the other Cavin localization detection. The work includes the structural determination of the nanobody with HR1 and is in substantial amount.

Comments for the author

Major points:
Whether the unfolding induced by the nanobody binding and the unfolding by the mutation are similar has not been clarified because the unfolded structure is invisible. Please address this point by the TEM of the protein itself or any other means, or add the discussion on that.
In the colocalization figures of nanobody and cavin1, the staining is not identical to those of Cavin1 signals. The reason for the difference will be upon the new finding on cavin 1 and caveolae biology and should be described.
The relevance of the unfolding of Cavin in caveolar function was not identified. It would be better if some indication of the caveolar function by the presence of the nanobody or unfolded phosphomimetic mutants.

Minor point
Figure 8, There are some rounded cells, and better pictures will be provided.

First revision

Author response to reviewers' comments

Reviewer 1: SUMMARY OF THE ADVANCE MADE IN THIS PAPER AND ITS POTENTIAL SIGNIFICANCE TO THE FIELD

In this manuscript, the authors, Gao et al., use nanobodies to characterize the structure of the helical region (HR1) of cavin1 in solution and bound to membranes and caveolae. They describe the development and characterization of these nanobodies, which could be very useful to the field. The study uses a combination of protein crystallization, biochemical analysis of reconstituted systems and microscopy analysis of proteins on caveolae in cells. They show that the helical bundling of the C-terminal region of the trimeric HR1 is relatively instable. Using the specificity of different nanobodies, they report that the C-terminal region does not form a helical coiled coil when associated with caveolae in agreement with previous modelling work. The experiments are sound and the conclusions are supported by the data. This is very interesting and important work that advance the caveolae field but also contributes to our structural understanding peripherally attached membrane proteins.

SUGGESTIONS TO AUTHORS
Suggestions for improvements:

1. The manuscript would majorly benefit from overall improvement of the representation of the data, figure outline and labelling. Check labeling, scale bar colors etc of all figures. Examples:
(i) It would be more intuitive to the reader if the ITC for A12 and B7 were kept to one color each throughout the paper.
We have now adjusted the colours for the ITC graphs for consistency throughout the manuscript as requested.

© 2025. Published by The Company of Biologists under the terms of the Creative Commons Attribution License (https://creativecommons.org/licenses/by/4.0/).

(ii) Figure 1F is referenced in the main text as 1G.
This has been corrected.

(iii) In the figure legend figure 1B is referenced as 1C and vice versa.
This has been corrected. – Ya-done

(iv) Fig 4A; Confusing labeling of input and Co-ip. In the figure legend it is stated that western blot was performed with antibodies against Cavin1 and Caveolin1, antibody against GFP should be included.
We thank the reviewer for pointing this out have altered the labelling of Fig. 4A to make it clearer.

(v) Fig 4 B-C; If possible, remove text from the scale bar in the images.
The text has been removed.

(vi) Fig 4B; In the main text it is stated that colocalization of the nanobodies and CAV1 in HeLa cells was tested but no such image exists within the manuscript.
This has been corrected.

(vii) Fig 4C; Why not show the colocalization of Cavin1 and the nanobodies as in B? Is there any particular reason for using the A431 cells, please specify in the main text.
We thank the reviewer for the question. In Panel 4C we decided to use an orthogonal set of both cells and caveola marker to provide additional confirmation of the co-localisation with caveolae.

(viii) Fig 4D; Are there any visible caveolae in the NbA12 image? Why is this image in a dashed line box? Make the figure cohesive with the NbB7 image. It is not clear from the legend which size the different scale bars have.
We have provided information on the scale bars in the updated Figure legend and have relabelled the NbA12 panel as Fig. 4E to more clearly distinguish this from the NbB7 images in Fig. 4D. Caveolae in the cells expressing NbA12-GFP that are not labelled by APEX-GBP are indicated with white arrows.

(ix) Fig 6; Remove the zoom out images (top images). Make the presentation of the profile analysis cohesive in terms of ticks on the Y-axis.
We have adjusted the line profile analyses to have a consistent Y axis across all panels and removed the zoomed-out images.

(x) Fig 8; Different sizes of the images are shown. Make A and B as in C and D.
We have resized each of the images to be the same size as Panel C, and as in Fig. 6 adjusted the line profile analyses to have a consistent Y axis across all panels.

2. The data in Figures 1-3 is about the characterization of the nanobodies and in my view, not all the ITC data needs to be in the main figures. If some of the data are moved to supplementary data, then I think it would be possible to have just one figure for the characterization of the nanobodies. In this way more focus will be put on the conclusions drawn by using the nanobodies.
We thank the reviewer for this suggestion. In answer we have moved all the previous Fig. 2 panels related to binding of the nanobodies to cavin homologues into Supplementary Fig. S1.

3. In Fig 1A the A4 nanobody is claimed to have affinity for GST-HR1. Yet, since the amount of A4 nanobody is much higher in also the supernatant, I doubt that this is a relevant signal. By instead evaluating the ratio of the pellet and total (pellet plus supernatant) for each nanobody, I find it more likely that it is only A12 and B7 that show significant binding. This would also be much more consistent with the ITC data following up on these nanobodies.
We thank the reviewer for this comment and have amended the text to reflect this uncertainty.

4. The authors have tested different Cavin1 mutants and state in the abstract that the conserved buried hydrogen-bonded His-Thr pair mutation reveals a selective regulation of Cavin2/Cavin3 association to Cavin1. This should be corrected in the abstract since they show that this mutation does not affect the localization of either Cavin2 or Cavin3. They do however show that it is the phosphomimic TS/DD mutation that impact the localization of Cavin2 to caveolae.
This text has been altered as requested.

© 2025. Published by The Company of Biologists under the terms of the Creative Commons Attribution License (https://creativecommons.org/licenses/by/4.0/).

5. In the results section the choice of cell lines for different experiments could be presented to the reader.
We have included additional text on line 160, Page 7.

6. I don't understand the statement starting on line 169 regarding Fig S1D. Lower expression levels of what? Specify if the NbB7-GFP is expressed in higher levels.
This refers to the lower expression levels of the GFP fusion proteins.

7. Reformulate statement line 284 "Caveolae formation and recruitment …."
By expression of only cavin1, cavin2 and cavin 3 cannot be rectruited if they are not expressed in the cells?
We thank the reviewer for pointing this out and have clarified the text here for the reader. In the experiments we performed required co-expression of Cavin1-GFP to recruit either Cavin2-mCherry or Cavin3-mCherry.

8. I don't understand what is meant by "plasma" on line 224 and 225? Plasma membrane?
This has been amended.

9. I would suggest to remove the sentence starting on line 278 since it is not required
We have decided to leave this sentence for now, as we believe it provides context for the reader at the beginning of the section as to what mutants are being examined in cells.

Reviewer 2: SUMMARY OF THE ADVANCE MADE IN THIS PAPER AND ITS POTENTIAL SIGNIFICANCE TO THE FIELD
This paper characterizes the nanobody binding to the Cavin1 HR1 domain. The nanobody binding would induce the unfolding of the part of Cavin1. The mutants of Cavin1 also exhibited an unfolded structure. The nanobody had a higher affinity to these mutants, suggesting the preference for the unfolded Cavin, which would reflect the differential staining by the antibodies in comparison with the other Cavin localization detection. The work includes the structural determination of the nanobody with HR1 and is in substantial amount.

SUGGESTIONS TO AUTHORS
Major points:
Whether the unfolding induced by the nanobody binding and the unfolding by the mutation are similar has not been clarified because the unfolded structure is invisible. Please address this point by the TEM of the protein itself or any other means, or add the discussion on that.
We appreciate the concerns of the reviewer, however, performing TEM analyses of the Cavin1 HR1 domain either alone or in complex with the nanobody is just not feasible given its small size. Moreover, EM analyses are not likely to be able to resolve whether disordered regions are the same or different. We argue that that whether the disorder in the C-terminal region of the HR1 domain is precisely the same between the nanobody-bound protein or the mutated protein is not critical to the work and will be a very difficult question to answer with any experimental approach. It is possible that MD simulations might provide some clues but it is unlikely to be able to provide a conclusive answer and lies outside the scope of this study.

In the colocalization figures of nanobody and cavin1, the staining is not identical to those of Cavin1 signals. The reason for the difference will be upon the new finding on cavin 1 and caveolae biology and should be described.
We apologise but we don’t fully understand what this question means. In all the imaging experiments we have reported the findings regarding nanobody colocalization with Cavin1 and CAV1 at caveolae in detail and with appropriate explanations where required. In images such as Fig. 4B and 4C, Fig. 6 and Fig. 8 we believe we have discussed the findings and their relevance to the ability of the nanobody or Cavin1 mutants to be recruited to cellular caveola structures.

The relevance of the unfolding of Cavin in caveolar function was not identified. It would be better if some indication of the caveolar function by the presence of the nanobody or unfolded phosphomimetic mutants.
As discussed throughout the text, previous studies have suggested that unfolding or structural plasticity in the C-terminal region of the Cavin1 HR1 domain is important for the ability of Cavin1

© 2025. Published by The Company of Biologists under the terms of the Creative Commons Attribution License (https://creativecommons.org/licenses/by/4.0/).

to associate with membranes. As our results show, the nanobody B7 has the capacity to bind this ‘unfolded’ form of the Cavin1 HR1 domain and supports this previous proposal. We also find that mutations in this region perturb its ability, in particular a phosphorylation mimicking mutation, can impact subsequent specific recruitment of Cavin2 and Cavin3.

Minor point
Figure 8, There are some rounded cells, and better pictures will be provided.
We thank the reviewer for pointing this out and have improved the presentation of the images in Fig. 8 accordingly.

Second decision letter

MS ID#: jcs.263756R1

MS Title: Cavin1 binding nanobodies reveal structural flexibility and regulated interactions of the N-terminal coiled-coil domain

Authors: Ya Gao; Vikas Tillu; Yeping Wu; James Rae; Thomas Hall; Kai-En Chen; Saroja Weeratunga; Qian Guo; Emma Livingstone; Wai-Hong Tham; Robert Parton; Brett Collins

Article Type: Research Article

Dear Dr Collins,

I hope the cyclone was not too horrific and I am happy to tell you that your manuscript has been accepted for publication in Journal of Cell Science, pending standard publication integrity checks.

© 2025. Published by The Company of Biologists under the terms of the Creative Commons Attribution License (https://creativecommons.org/licenses/by/4.0/).
